# (*S*,*S*)‑5-Chloro-3-((3,5-dimethylphenyl)sulfonyl)‑*N*‑(1-oxo-1-((1-(pyridin-4-yl)ethyl)amino)propan-2-yl)‑1*H*‑indole-2-carboxamide, a New Dishevelled 1 and P‑Glycoprotein Dual Inhibitor as a Anticancer Agent

**DOI:** 10.1021/acs.jmedchem.6c00706

**Published:** 2026-06-12

**Authors:** Michela Puxeddu, Zeyu Cui, Claudia Colla, Marianna Nalli, Simone Manetto, Alessia Ciogli, Petra Cuřínová, Marianna Bufano, Angelo Toto, Stefano Gianni, Arianna Pastore, Mariano Stornaiuolo, Enke Baldini, Salvatore Ulisse, Joanna Kopecka, Chiara Riganti, Chiara Bigogno, Giulio Dondio, Te Liu, Antonio Coluccia, Giuseppe La Regina, Romano Silvestri

**Affiliations:** a Laboratory Affiliated with the Institute Pasteur Italy - Cenci Bolognetti Foundation, Department of Drug Chemistry and Technologies, 9311 Sapienza University of Rome, Piazzale Aldo Moro 5, Rome 00185, Italy; b Shanghai Geriatric Institute of Chinese Medicine, 66322 Shanghai University of Traditional Chinese Medicine, 365 South Xiangyang Road, Shanghai 200031, China; c Department of Organic Chemistry, 52735 University of Chemistry and Technology Prague, Technická 5, Prague 6 16628, Czech Republic; d Laboratory Affiliated with the Institute Pasteur Italy - Cenci Bolognetti Foundation, Biochemical Sciences “Rossi Fanelli”, Institute of Biology and Molecular Pathology of CNR, Sapienza Università di Roma, Piazzale Aldo Moro 5, Rome 00185, Italy; e Department of Pharmacy, University of Naples “Federico II”, Via Domenico Montesano, 49, Naples 80131, Italy; f Department of Surgery, 9311 Sapienza University of Rome, Viale Regina Elena 324, Rome 00185, Italy; g Department of Oncology and Molecular Biotechnology Center ″Guido Tarone″, Via Nizza 44, Turin 10126, Italy; h Aphad SrL, Via della Resistenza 65, Buccinasco 20090, Italy

## Abstract

Dishevelled (DVL) proteins are key mediators of the Wnt/β-catenin signaling pathway, involved in signal transduction from membrane receptors to intracellular effectors. DVL up-regulation has often been correlated with tumor progression and metastasis. DVL1 was found overexpressed in multidrug-resistant colorectal cancer (CRC) cells. Here, we describe the synthesis of new indole-2-carboxamides **2–18** as DVL1 inhibitors. Compound (*S*,*S*)-**15** showed potent DVL1 inhibition with IC_50_ of 0.97 ± 0.21 μM and specific binding to the DVL1 PDZ domain. (*S*,*S*)-**15** strongly reduced β-catenin expression in HCT116 CRC cells and significantly decreased tumor volume and weight in a xenograft model. Additionally, (*S*,*S*)-**15** inhibited P-glycoprotein (P-gp), restoring sensitivity to doxorubicin (DOX) in HT29/DX CRC chemoresistant cells. (*S*,*S*)-**15** demonstrated high metabolic stability in human liver microsomes, and an acceptable pharmacokinetic profile following IV administration in mice. Our findings indicate that compound (*S*,*S*)-**15** represents a promising dual-targeting antitumor candidate for CRC treatment.

## Graphical abstract

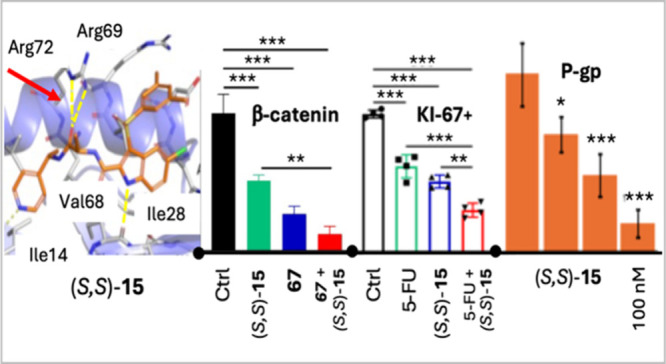

## Introduction

The wingless/integrase-1 (Wnt)/β-catenin pathway has been shown to play a critical role in the oncogenic processes since it regulates the translocation of β-catenin in the cell nucleus.^1^ Aberrant Wnt signaling was observed in many cancer entities.^2^ Experimental evidence for a critical role of Wnt in human cancer was described by Polakis.^3^ The Wnt pathway was also associated with cancer stem cell generation^4^ and variation^5^ during cancer progression. Advances in knowledge on tumor initiation and growth validated the Wnt/β-catenin signaling pathway as a promising target for tumor treatment,^6^ especially in colorectal cancer (CRC).^7^ The complexity of the signaling network and the multitude of proteins involved in the Wnt cascade have been a major obstacle to the development of finely tuning drugs.^8^ Therefore, a substantial proportion of the overall Wnt/β-catenin targeting agents fail to progress beyond the preclinical stage.^9^

In the canonical Wnt pathway, recruitment of Dishevelled (Dsh/DVL) to Frizzled (FZ) receptors and of Axin to LRP5/6 coreceptors prevents the constitutive degradation of cytosolic β-catenin.^10^ As a result, accumulated β-catenin moves into the cell nucleus where it activates the lymphoid enhancer-binding factor (LEF) and T-cell factor (TCF) transcription factors. LEF/TCF activation triggers transcription of Wnt target genes, like *cyclin D1*, *c-Myc*, and *lgr5*, that are responsible for the development and progression of several solid tumors and hematological malignancies.^11^

DVL proteins regulate pathway-specific subcellular complexes that ensure correct signal propagation within the cell.^12^ The DVL protein family comprises three paralogues (DVL1, 2, and 3), sharing 59–67% amino acid (aa) sequence identity^13^ and characterized by three highly conserved domains in humans and mice: (i) the 82–85 aa *N*-terminal DIX (Dishevelled–Axin) domain, which promotes DVL oligomerization and binding to Axin; (ii) the 90 aa PDZ (postsynaptic density 95/disc large/zonula occludens-1) domain, implicated in multiprotein complex formation, and (iii) the 80 aa carboxyl-terminal DEP (Dvl, Egl-10, and Pleckstrin) domain, which facilitates the recruitment of DVL to the plasma membrane.^14^ Two additional conserved regions are involved in protein–protein interaction and/or phosphorylation.^10^

The PDZ domain is characterized by a classical two α-helical (α1 and α2) and six β-strand (β1 to β6) structure. It mediates the assembly of protein complexes (e.g., AMPA, NMDA, and G-protein coupled receptors) involved in signal transduction, cell–cell junction, and cell-matrix adhesion.^15^ Due to its key role in DVL function, the PDZ domain has become an attractive therapeutic druggable targets.^16^ DVL overexpression potently activates the Wnt/β-catenin signaling.^10^ In tumor cells, DVL1 and DVL3 can translocate into the nucleus, where they bind to β-catenin, promote nuclear complex formation and transcriptional activity, and increase the levels of multidrug resistance proteins, without significant effect on accumulation of cytoplasmic β-catenin.^17^ The DVLs are often correlated with cancer cell proliferation, tumor progression, and metastasis.^18^ DVL1 and DVL3 were found to be overexpressed in multidrug-resistant (MDR) CRC cells (HCT-8/VCR).^17^

Our research focus is on identifying small-molecule modulators of the Wnt pathway. In previous works, we described a negative modulator of FZD4 (a member of the FZD family) binding at an allosteric site within the intracellular loop 3 (ICL3),^19^ and a positive allosteric modulator obtained by fine-tuning of the same scaffold.^20^ Also, we identified a highly selective inhibitor of Na^+^/H^+^ exchanger 3 regulating factor 1 (NHERF1) targeting the same C-terminal region used by FZDs to form contacts with DVL through the PDZ domains.^21^

Recently, structure-based virtual screening (VS) studies aimed at finding DVL1 inhibitors having minimal effect on NHERF1 led to the successful identification of compound **1** (Chart 1) as a potent and selective inhibitor of DVL1. The pure (*S*)-**1** enantiomer showed greater inhibition of DVL1 than the (*R*)-enantiomer. Binding competition assays and biological evaluation in cells confirmed its mechanism of action, namely, the impairment of the Wnt pathway and the resulting anticancer activity.^22^ The six-membered terminal ring and the (*S*) configuration of carbon at position 3 of the side chain were key structural features for the binding to DVL1 (Chart 1). However, little was known about the role played by other substitutions. Thus, we synthesized compounds **2–18** (Table 1) to expand the structure–activity relationship (SAR) studies. We focused on (*i*) position 5 of the indole nucleus, (*ii*) positions 3,5 of the phenyl ring, (*iii*) bridging group, (*iv*) position 5 of the side chain, and (*v*) the terminal pyridinyl group.

**Chart 1:**
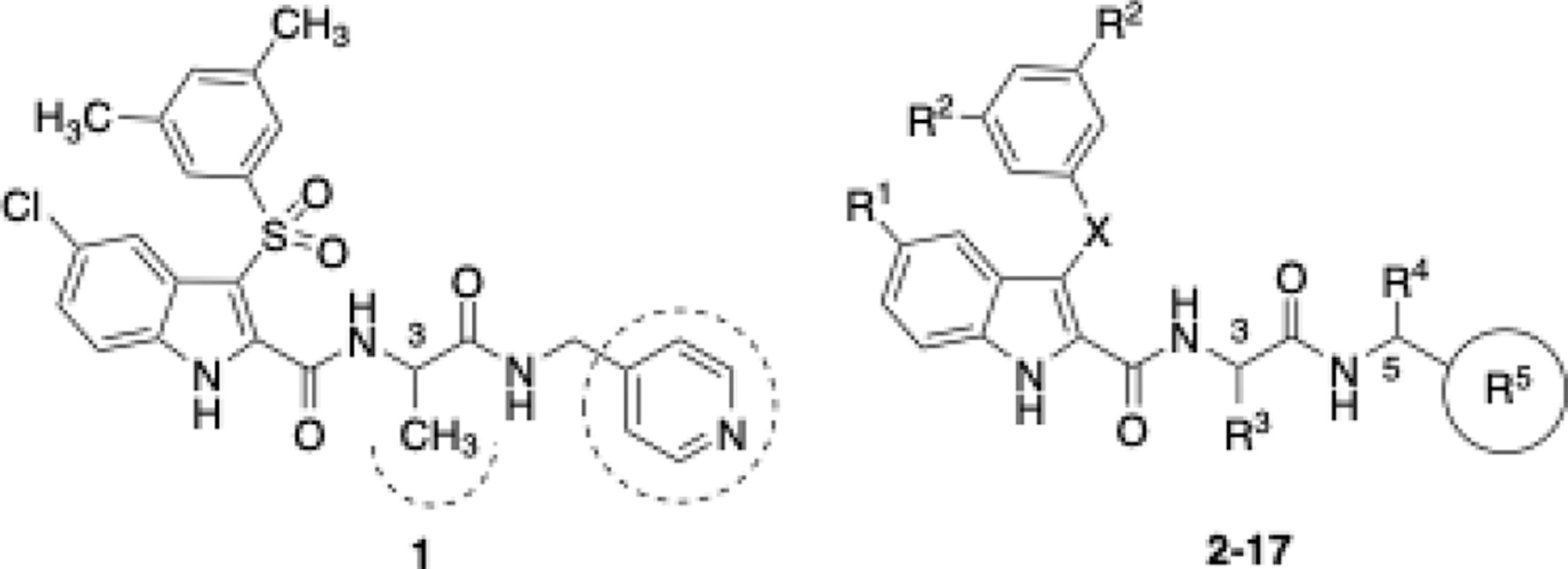
Key Structural Features of Compound **1** for the Binding to DVL1 PDZ Highlighted by Dotted Line^a^

a General structure of new compounds **2**–**17** for SAR studies; see Table 1 for R_1_–R_5_ and X values.

**Table 1:**
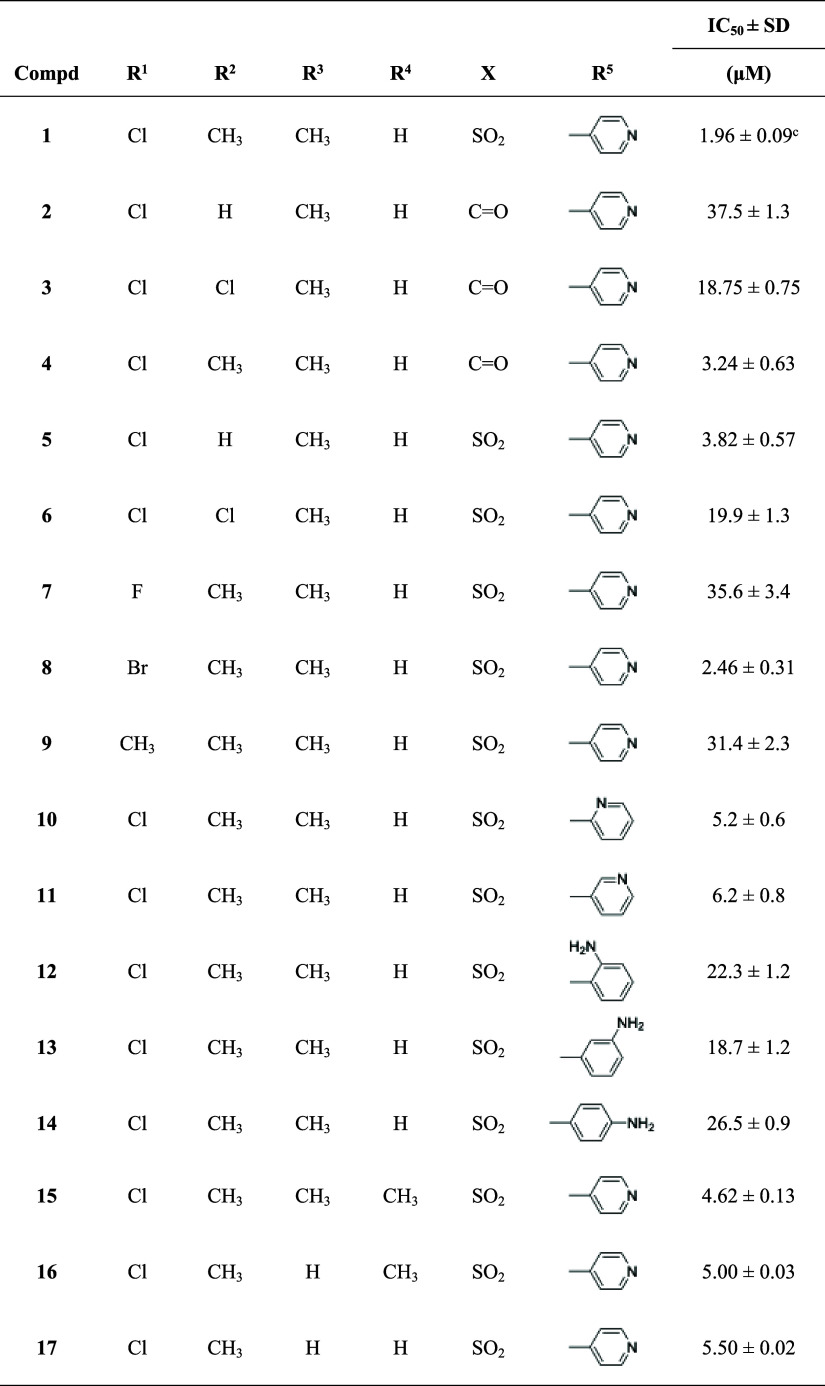
DVL1 Binding Inhibition by Compounds **1**–**17** ^a^ ^,^ ^b^

a Inhibition of DVL1 binding. Experiments were performed in triplicate.

b Compounds **1–14** and **16** are racemates with one stereocenter, **15** is a diastereomeric mixture containing two stereocenters, and **17** has no stereocenter.

c Reassayed.^22^

We studied ADME (Absorption, Distribution, Metabolism, Excretion) properties, since results of phase I oxidative metabolism of (*S*)-**1** were unsatisfactory. In silico ADME and metabolic liability predictions indicated a favorable profile for compound **15** (as mixture of diasteroisomers), with improved gastrointestinal absorption predicted by SwissADME and reduced metabolic liability suggested by SMARTCyp relative to compound **1**. The four enantiomers of **15** were separated by enantioselective HPLC. We tested the efficacy of (*S*,*S*)-**15** in inhibiting DVL1, modulating β-catenin expression, and exerting antitumor activity across a panel of CRC cell lines. Furthermore, we evaluated its ability to restore doxorubicin (DOX) sensitivity in resistant CRC cells and, ultimately, to impair tumor growth in xenograft models.

## Results and Discussion

### Synthesis

Compounds **2–11** and **15–20** (Scheme 1) were obtained by treating **21–26**, **50**, **55**, **60**, and **66** with an appropriate amine, 1-[bis­(dimethylamino)­methylene]-1*H*-1,2,3-triazolo­[4,5-*b*]­pyridinium 3-oxid hexafluorophosphate (HATU) and *N*,*N*-diisopropylethylamine (DIPEA) in *N,N*-dimethylformamide at 25 °C for 12 h under an argon stream. The use of HATU and DIPEA in place of benzotriazol-1-yloxytripyrrolidinophosphonium hexafluorophosphate (PyBOP) and triethylamine (Et_3_N) has improved yields compared to the previously described reaction conditions.^23^ Compounds **12–14** were obtained by Tin­(II) chloride reduction of derivatives **18–20** in ethyl acetate at 80 °C for 3 h. Compounds **21–26**, **50**, **55**, **60**, and **66** were synthesized by lithium hydroxide hydrolysis of derivatives **27–31**, **49**, **54**, **59**, and **65** in aqueous tetrahydrofuran at 25 °C for 3 h (Scheme 1).

**Scheme 1:**
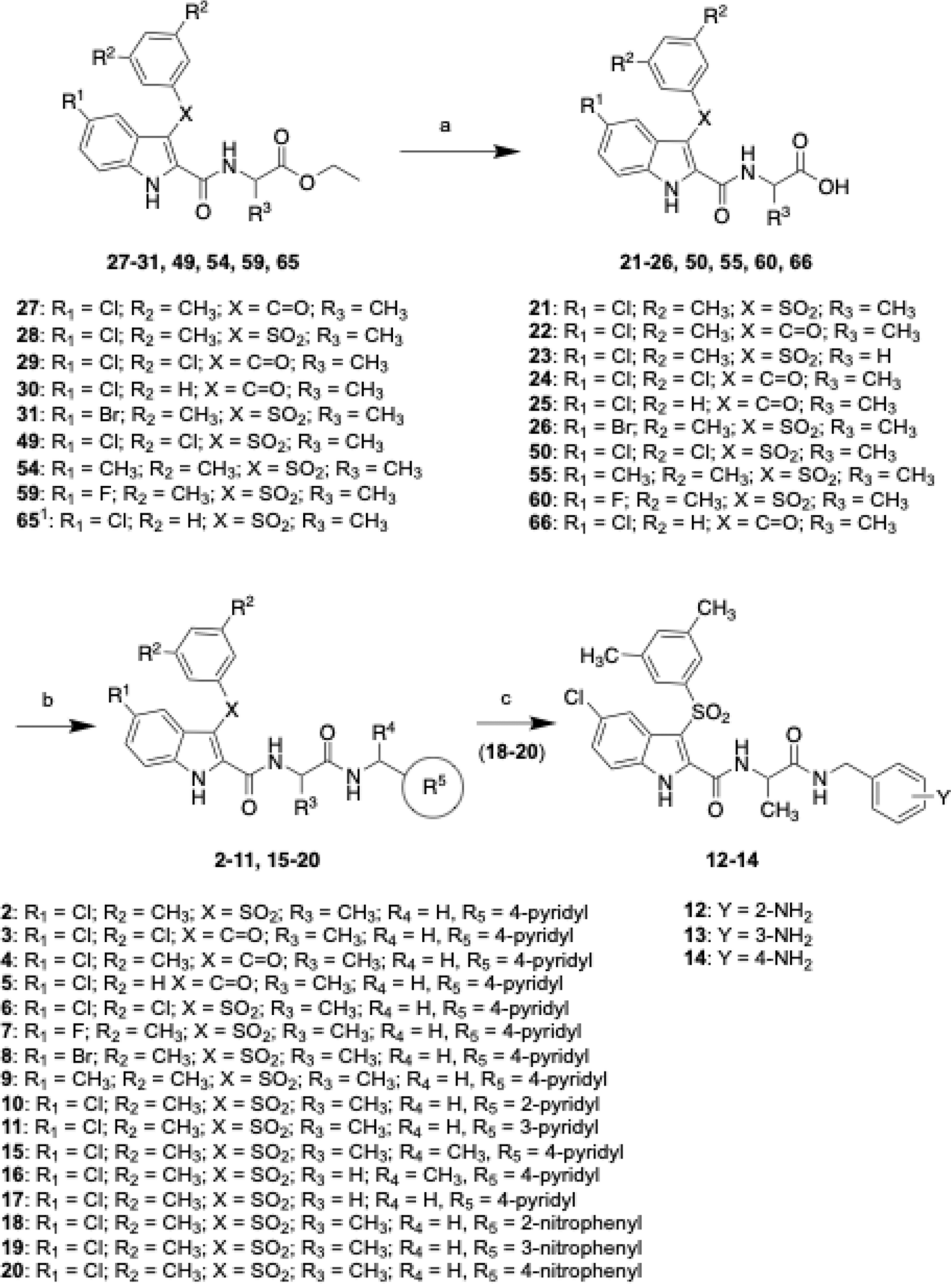
Synthesis of Compounds **2**–**20** [Fn sch1-fn1]

a
*Reagents and reaction conditions:* (a) LiOH, THF, water, 25 °C, 3 h, 58–91%; (b) appropriate amine, DIPEA, HATU, dry DMF, Ar, 25 °C, 12 h, 50–98%; (c) tin­(II) chloride dihydrate, AcOEt, 80 °C, 3 h, 47–87%. ^1^ **65** was used as a methyl ester.

Intermediate compounds **27–31**, **49**, **54**, **59**, and **65** were prepared by coupling reaction of acids **32–36**, **48**, **53**, **58**, and **64** with (*rac*)-ethyl alaninate under the same conditions used for the synthesis of **2–11** and **15–20** (Scheme 2). Compounds **32–36**, **48**, **53**, **58**, and **64** were obtained by hydrolysis in 3 N sodium hydroxide and ethanol of esters **37–40**, **42**, **47**,**52**, **57** and **63** at 80 °C for 1 h. Sulfur derivatives **41**, **43**, **46**, **51**, **56**, and **62** were converted to the corresponding sulfones **38**, **42**, **47**, **52**, **57**, and **63** by stirring with *meta*-chloroperoxybenzoic acid (mCPBA) in ice-cooled dichloromethane and then at 25 °C for 3 h. Compounds **37**, **39**, and **40** were obtained by microwave reaction, close vessel mode, of an appropriate benzoyl chloride with ethyl 5-chloro-1*H*-indole-2-carboxylate in the presence of anhydrous aluminum chloride in dichloroethane while stirring at 110 °C for 2 min. Compounds **46** and **56** were prepared by reaction of the 1,2-bis­(diaryl)­disulfane **45** and **44**, respectively, with the appropriate indole-2-carboxylate in the presence of potassium carbonate in DMSO at 100 °C for 9 h. Compounds **41**, **43**, **44**, **45**, **51**, **62**, and **62** were synthesized as previously reported (see the Supporting Information).

**Scheme 2:**
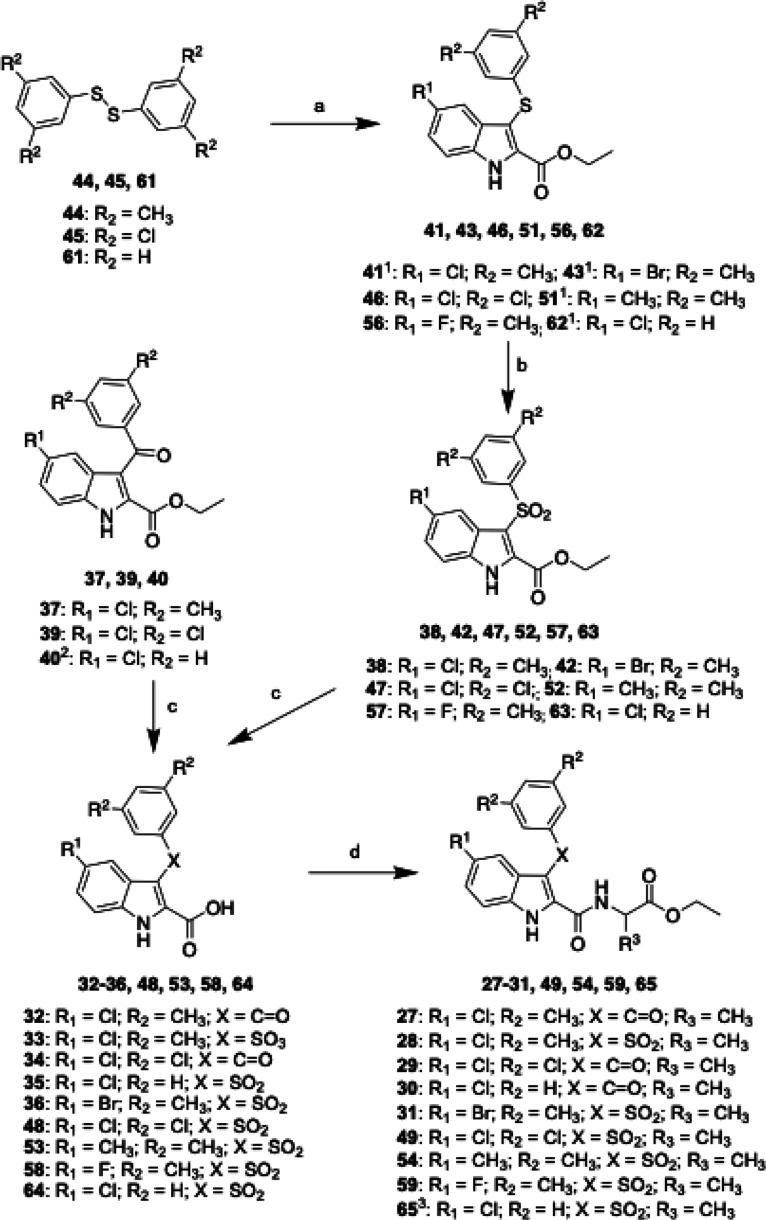
Synthesis of Intermediate Derivatives[Fn sch2-fn1]

a
*Reagents and reaction conditions:* (a) **46** and **56**: appropriate indole, K_2_CO_3_, DMSO, 100 °C, 9 h, 60–73%; (b) mCPBA, dichloromethane, 0–25 °C, 3 h, 32–71%; (c) NaOH, ethanol, 80 °C, 1 h, 70–100%; (d) (e) (*rac*)-ethyl alaninate, DIPEA, HATU, dry DMF, Ar, 25 °C, 12 h, 48–80%. ^1^ **41**, **43**, **51**, and **62**: see the Supporting Information; ^2^ **37**, **39**, and **40** were obtained from appropriate indole and benzoyl chloride, AlCl_3_, DCE, and MW (close vessel) 25 to 110 °C, 2 min, 32–68%. ^3^ **65** was synthesized as a methyl ester.

### DVL1 Inhibition

IC_50_ values for the inhibition of DVL1 binding to a peptide mimicking the C-terminal portion of TMEM88 by **2–17** and reference compound **1** are shown in Table 1. All compounds were tested as racemates except **15** that was evaluated as a mixture of four diastereoisomers, bearing two stereocenters, and **17** that has no stereocenter. The substituent at position 5 of the indole played a crucial role for the binding. Replacement of the 5-chlorine of **1** (IC_50_ = 1.96 μM) with a bromine atom to give **8** (IC_50_ = 2.46 μM) slightly reduced (1.3-fold) the DVL1 binding inhibition, whereas introduction of a fluorine atom (**7**) or a methyl group (**9**) caused a dramatic drop of activity. Replacement of the sulfonyl bridging group of **1** with a carbonyl group to give **4** (IC_50_ = 3.24 μM) caused 1.7-fold reduction of activity. Independently on the nature of the bridging group, the strongest DVL1 inhibition correlated with the presence of methyl groups at positions 3 and 5 of the outer phenyl ring (compare **1** with **4**, IC_50_ = 3.4 μM). In both sulfonyl and carbonyl series, the 3,5-dichlophenyl substitution pattern diminished the DVL1 inhibition (compare **3** with **6**). In the absence of substituents (R_2_ = H), sulfone **5** (IC_50_ = 3.8 μM) inhibited stronger DVL1 than the carbonyl counterpart **2** (IC_50_ = 37.5 μM). Replacement of the terminal pyrimidin-4-yl of **1** with the isomeric pyrimidin-2-yl (**10**, IC_50_ = 5.2 μM) or pyrimidin-3-yl (**11**, IC_50_ = 6.2 μM) rings reduced DVL1 inhibition by 2.7- and 3.2-fold, respectively. Extrusion of the pyridine nitrogens of **1**, **10**, and **11** to give anilines **12–14** caused a dramatic drop of activity. We synthesized compounds **15–17** to evaluate the impact played by methyl group(s) at the side chain. Compound **15** bearing two methyl groups at positions 3 and 5 inhibited the DVL1 binding with IC_50_ of 4.6 μM. Both **16** (IC_50_ = 5.0 μM) and **17** (IC_50_ = 5.5 μM) lacking the methyl at position 3 of the side chain were less effective. In agreement with our previous studies,^24–26^ sulfur and methylene derivatives showed weak DVL1 inhibition. A SAR summary of DVL1 inhibitory activities by compounds **1–17** is shown in Chart 2.

**Chart 2:**
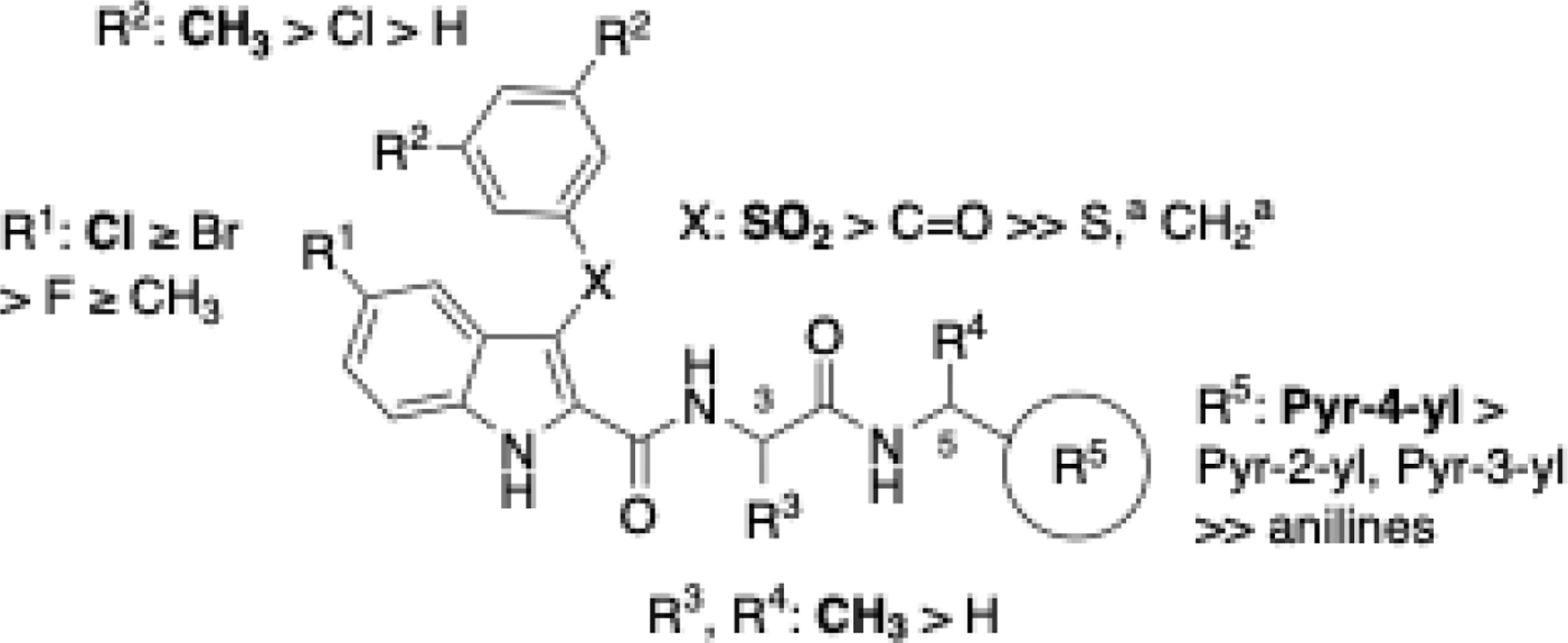
SAR Summary of Effects of R_1_–R_5_ and X on Inhibition of DVL1 Binding by Racemic Mixtures **1**–**16**, and **17** without a Stereocenter^b^

a Results not shown.
b The most effective atom/group is highlighted in bold font.

### Swiss-ADME Prediction

Physicochemical properties of racemates **1**, **8**, **15**, **16**, and compound **17** were predicted by the Swiss-ADME server^27^ (Table 2). Compound **1** and its bromo analog **8** were predicted to have quite similar % values of gastrointestinal absorption (GIA) after oral administration. Compound **15**, bearing two methyl groups at positions 3 and 5 of the side chain, showed the highest GIA value of 84.5%, while compounds **16** and **17**, lacking one and both methyl groups showed lower GIA values of 79.9 and 74.7%, respectively. A general correlation between LogP and GIA values was observed. Interestingly, all compounds were predicted to have low potential toxicity in preclinical studies and did not yield alert for potential pan-assay interference structures (PAINS) (data not shown).^28^

**Table 2:** Physicochemical Properties of Compounds **1**, **8**, **15**, **16**, and **17** Predicted by the Swiss-ADME Server^a^

compd	MW^b^	**LogP** ^c^	**LogSw** ^d^	**tPSA** ^e^	**GIA** ^f^ **(%)**	**Caco2** ^g^	**Rot** ^h^	**Lr** ^i^	**Vr** ^j^	3/75^k^
**1**	525.02	3.98	–5.44	129.40	79.7	high	9	1	0	low
**8**	569.47	4.05	–5.76	129.40	77.4	high	9	1	0	low
**15**	539.05	4.38	–5.77	129.40	84.5	high	9	1	0	low
**16**	525.02	3.98	–5.44	129.40	79.9	high	9	1	0	low
**17**	510.99	3.58	–5.11	129.40	74.7	high	9	1	0	low

a Physicochemical properties predicted by Swiss-ADME server (24).

b Molecular weight.

c Octanol–water partition coefficient predictor by the XLOGP3 method.

d Logarithm of a drug’s solubility in water.

e Topological polar surface area.

f GIA, gastrointestinal absorption.

g Apparent Caco2 permeability.

h Rotatable bonds.

i Lipinski rule violation.^29^

j Veber rule violation.^30^

k Potential toxicity in preclinical studies.

### SMARTCyp Prediction

SMARTCyp is an in-silico method that predicts the sites of cytochrome P450-mediated metabolism of drug-like molecules.^31^ Originally developed for CYP3A4, it also provides reliable predictions for CYP2D6 and CYP2C9 isoforms. We are aware that the chirality can greatly affect the metabolic stability. Despite these limitations, we used SMARTCyp to get some indications of the metabolic liability of racemate **1** and diastereomeric mixture **15**.^32^ The software ranks the atoms by score correlating the lowest score to the highest probability of being a site of metabolism. Compound **1** exhibited lower score than **15**, a difference that may be attributed to the increased steric bulk introduced by the substituent on the side chain, likely due to reduced CYP3A4 accessibility and thereby enhanced metabolic stability. The top ranked CYP 3A4 metabolic sites of metabolism for **1** and **15** are shown in Chart 3 and Table 3.

**Chart 3:**
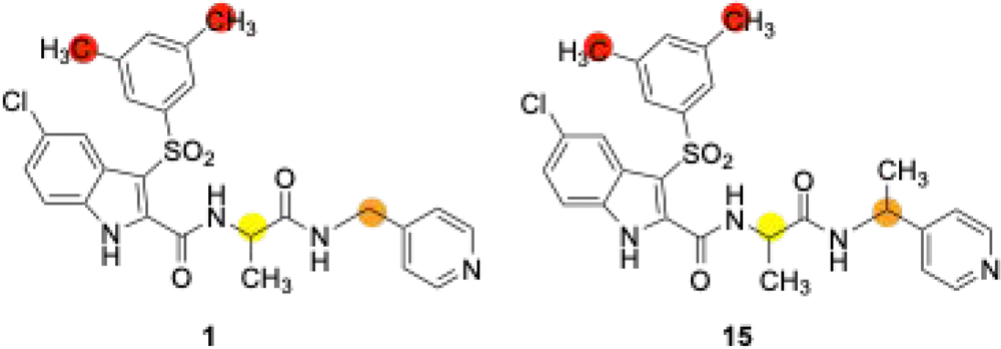
SMARTCyp Top-Ranked Metabolism Sites for Cytochrome P450 3A4-Mediated Metabolism^a^

a The highest probability of being a site of metabolism is highlighted by color circles in decreasing order: red, orange, yellow.

**Table 3:**
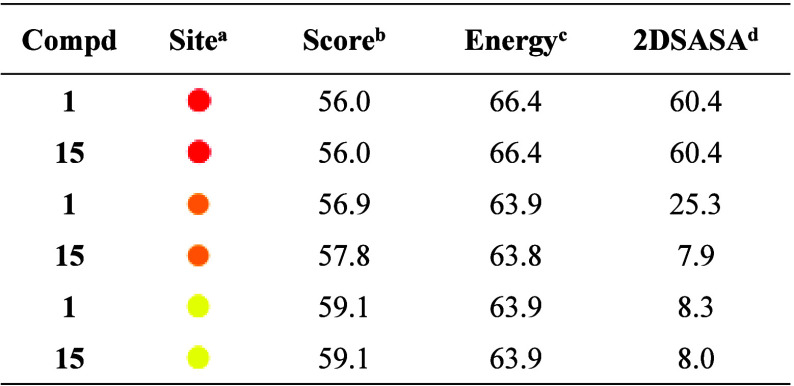
Score, Energy, and 2DSASA Values Predicted by SMARTCyp for **1** and **15**

a Top-ranking atoms are highlighted in Chart 3.

b Ranked score calculated by SMARTCyp.

c Energy (kJ/mol): approximate activation energy for the reaction of the catalytic site of a CYP with the molecule at this atom.

d 2DSASA: solvent-accessible surface area describes the local accessibility of an atom and is computed using the 2DSASA algorithm.

### Enantiomer Separation of Compound **15**

Due to the potential drug-like ability of compound **15**, we separated the four stereoisomers by enantioselective HPLC aiming to select the most active one. After semipreparative HPLC, the off-line ECD spectra were acquired for each stereoisomer, and by comparing the recorded ECD profiles, we identified the A-B and C–D as enantiomeric pairs (Figures 1 and 2, respectively). The enantiomeric excess was then calculated before proceeding with the next biological tests (Table 4).

**Figure 1:**
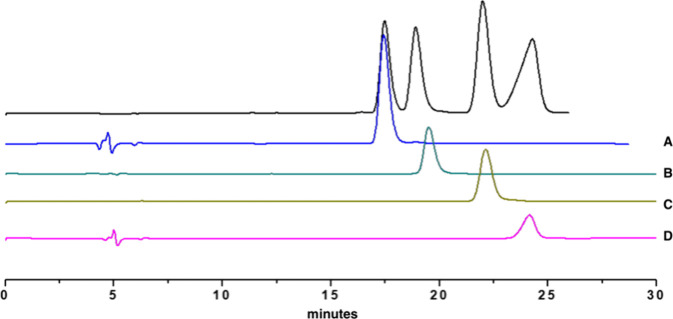
Enantioselective HPLC of **15**. Top: all resolved stereoisomers. Colored traces show the analytical control of single isolated stereoisomer after semipreparative HPLC.

**Figure 2:**
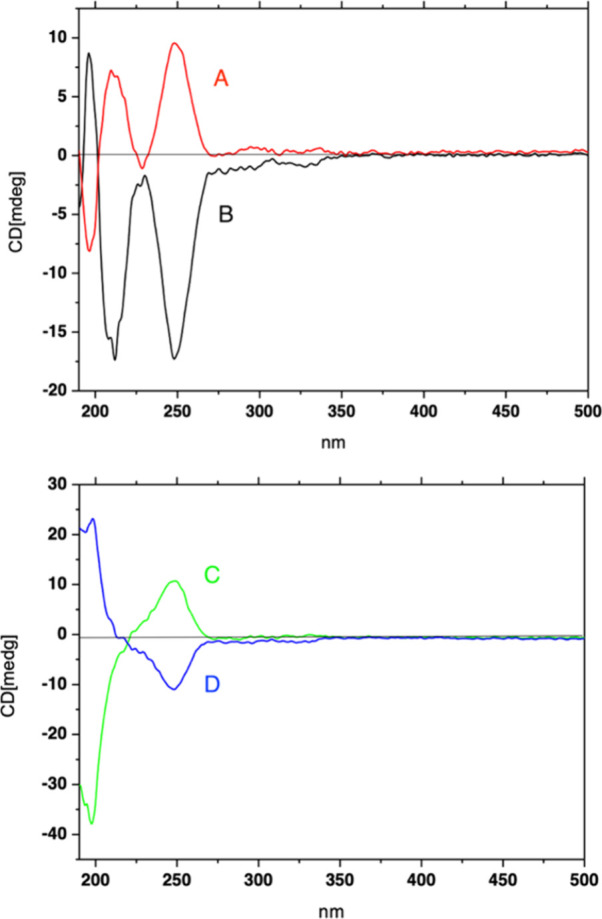
Off-line CD spectra of single stereoisomer. The mirror-image circular dichroism profiles match with the enantiomeric A/B and C/D pairs.

**Table 4:** DVL1 Binding Inhibition, Enantiomeric Excess, and Optical Rotation by Pure Stereoisomers of **15**

	IC_50_ ± SD^a^		e.e.^b^	alfaD^c^
**compd 15**	**(μM)**	**stereoisomer**	**(%)**	**(°)**
racemic	4.62 ± 0.13			
fraction A	4.34 ± 0.28	*S*,*R*	99.02	+35.15
fraction B	1.34 ± 0.16	*R*,*S*	99.99	–35.73
fraction C	0.97 ± 0.21	*S*,*S*	98.04	+27.13
fraction D	4.95 ± 0.36	*R*,*R*	98.10	–27.18

a Inhibition of DVL1 binding. Experiments were performed in triplicate.

b Enantiomeric excess.

c Specific optical rotation; solvent: CHCl_3_; c: A, 0.037, B, 0.033; C, 0.033, D, 0.031 (% g/mL).

The pure enantiomers A–D were assayed in vitro. As a DVL1 inhibitor, the C fraction gave the best IC_50_ value of 0.97 ± 0.21 μM (Table 4). Considering that the (*S*)-**1** precursor showed great inhibition of DVL1,^22^ we synthesized the diastereomeric samples using in turn the (*S*)- and the (*R*)-1-(pyridin-4-yl)­ethanamine as reported in Scheme 3 for the stereoisomer (*S*,*S*)-**15**. By enantioselective HPLC we attested that fraction C was the stereoisomer (*S*,*S*)-**15** and that the synthetic procedure preserved the configuration of stereocenters (less than 2% of diastereoisomer was detected, 1 Figure 1S).

**Scheme 3:**
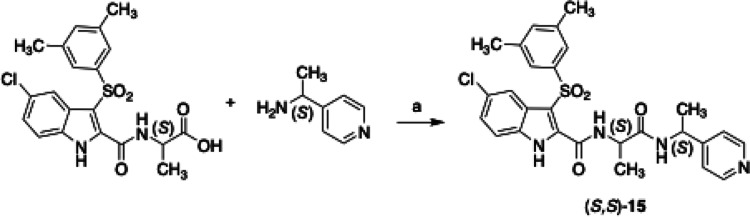
Synthesis of Enantiopure Compound (*S*,*S*)-**15** (Fraction C)[Fn sch3-fn1]

a
*Reagents and reaction conditions:* DIPEA, HATU, dry DMF, Ar, 25 °C, 12 h.

### Molecular Modeling Studies

To rationalize the observed enantioselectivity and gain molecular insight into the binding determinants, molecular docking studies were subsequently performed to investigate the binding modes of the individual enantiomers within the DVL1 PDZ domain. The 3,5-dimethylphenyl moiety engaged in π–cation interactions with Arg69, while the indole ring established hydrophobic contacts with Ile 28 and Val68. The pyridine ring was positioned within a hydrophobic pocket formed by Leu12 and Val75. Two hydrogen bonds were observed in all the enantiomers between the indole NH group and the pyridine nitrogen with the backbone carbonyls of Ile16 and Leu12, respectively (Figure 3). Importantly, the (*S*,*S*)-enantiomer formed additional hydrogen bonds with the side chain of Arg72, resulting in a more favorable interaction network within the PDZ binding groove (Figure 3A). These additional polar contacts likely contribute to the enhanced stability of the (*S*,*S*)-**15**/DVL1 PDZ complex. Overall, binding pose analysis and biological data confirmed the key role played by the (*S*) chiral center at C3,^22^ whereas the more permissive subpocket occupied by the C5 showed to tolerate both (*R*) and (*S*) configurations.

**Figure 3:**
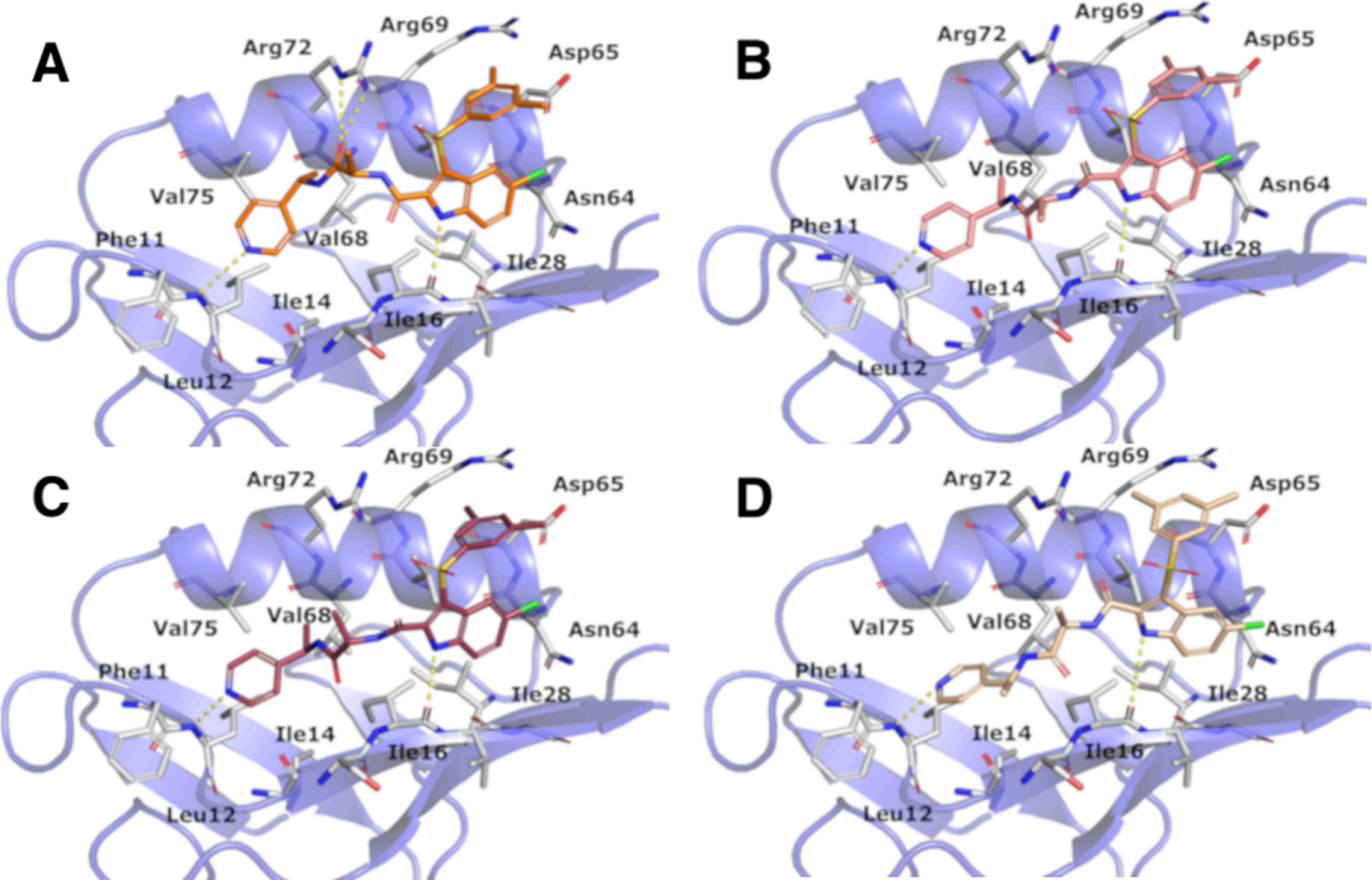
Docking poses of the four enantiomers of compound **15** in complex with the DVL1 PDZ binding site. (A) (*S*,*S*)-**15**: orange, (B) (*R*,*R*)-**15**: light pink; (C) (*S*,*R*)-**15**: dark pink; (D) (*R*,*S*)-**15**: beige. Residues involved in interactions: white sticks; the PDZ domain: light purple cartoon; H-bonds: yellow dotted lines. PDB IDs 3CBZ, 3CBY, and 3CC0 were used to generate the PDZ domain of DVL1.

### PDZ Binding Inhibition

Equilibrium binding experiments were conducted between the DVL1 PDZ domain and a peptide mimicking the C-terminal portion of TMEM88 protein (TSGKVWV) dansylated at its *N*-terminus. Experiments were performed by following the change in fluorescence emission at 510 nm (dansyl group), in the absence, and in the presence of compound (*S*,*S*)-**15** at 5 and 10 μM concentrations. The obtained data are reported in Figure 4. It is possible to observe that, at the concentration of 5 μM compound, (*S*,*S*)-**15** did not abolish the binding between PDZ and TMEM88, while at 10 μM, (*S*,*S*)-**15** produced a clear effect on the binding process. In fact, while in the absence of ligand and in the presence of 5 μM (*S*,*S*)-**15** the data could be reliably fitted with a hyperbolic equation, allowing calculation of a *K*_D_ of 11 ± 1 μM, in the presence of 10 μM (*S*,*S*)-**15**, data could not be fitted, suggesting inhibition of the binding event.

**Figure 4:**
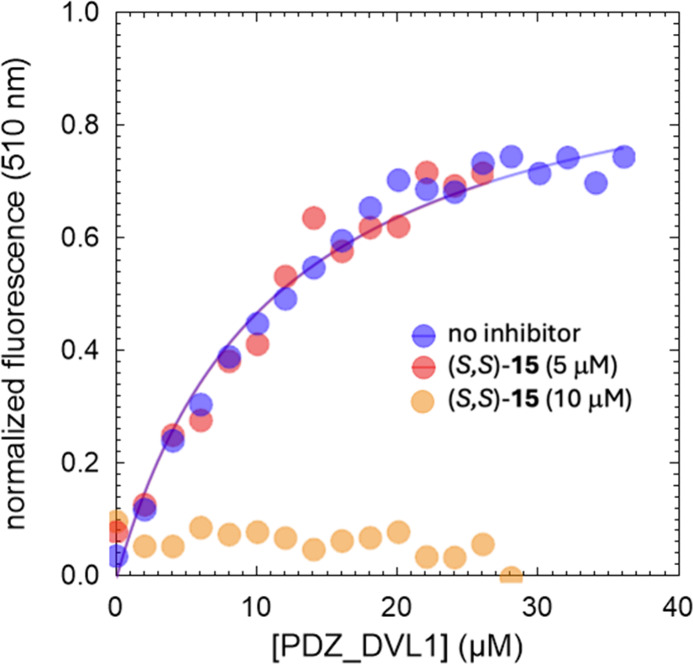
Equilibrium binding experiment between the PDZ domain of DVL1 and the C-terminal portion of TMEM88 in the absence and presence of (*S*,*S*)-**15** at 5 μM (red circles) and 10 μM (orange circles). Lines are the best fit for a hyperbolic function.

### Metabolic Stability

The metabolic stability of compounds (*S*)-**1** and (*S*,*S*)-**15** to phase I oxidative metabolism was assessed using human and mouse liver microsomes and verapamil as control compound (Tables 5 and 6). After incubation with human liver microsomes, compounds (*S*)-**1** and (*S*,*S*)-**15** showed intrinsic clearance values of 46.6 and 4.7 μL/min/mg protein, respectively, and in mouse liver microsomes, the values were 68.2 and 15.2 μL/min/mg protein, respectively. Compound (*S*,*S*)-**15** demonstrated superior metabolic stability profile compared to (*S*)-**1** in both human and mouse liver microsomes; in particular, (*S*,*S*)-**15** was 9.9-fold more stable than (*S*)-**1** in the human liver microsomes. LC–MS/MS analyses were carried out using an ESI (+) interface in multiple reaction monitoring (MRM) mode. Conditions and MRM transitions applied to the compounds are described in [Table tbl1] Table 1S, Supporting Information. The metabolic stability observed in the presence of human liver microsome enzymes encouraged to select compound (*S*,*S*)-**15** for further studies as a anticancer agent.

**Table 5:** *In Vitro* Determination of the Metabolic Stability after Incubation with Mouse and Human Liver Microsomes^a^

	human liver microsomes (HLM)		mouse liver microsomes (MLM)	
	μL/min/mg protein	min	μL/min/mg protein	min
**compd**	**Cli**±**SD**	***t*_1/2_** ±**SD**	**Cli**±**SD**	***t*_1/2_** ±**SD**
(*S*)-**1**	44.6 ± 10.3	32.0 ± 7.4	68.2 ± 11.7	20.6 ± 3.6
(*S,S*)-**15**	4.7 ± 0.4	297.3 ± 27.0	15.2 ± 1.3	91.5 ± 7.9
verapamil	210.6 ± 0.8	6.6 ± 0.0	291.2 ± 5.2	4.8 ± 0.1

a Results are expressed as the mean ± SD, *n* = 2. The standard compound verapamil showed metabolic stability in agreement with the literature^33^ and internal validation data.

**Table 6:** *In Vitro* Clearance Classification^a^

	Cli (μL/min/mg)		
classification	low Cli	medium Cli	high Cli
mouse	≤2.5	2.5–66	>66
human	≤1.8	1.8–48	>48

a Data obtained from refs 34–36 .

### Cell Growth Inhibition

The effects of compounds (*S*)-**1** and (*S*,*S*)-**15** on cell proliferation were tested in parallel on four human colon cancer cell lines, namely, SW620, SW480, HCT116, and DLD-1. Dose–response experiments were performed with a drug concentration range of 1–500 μM for 72 h, after which cell viability was measured by colorimetric assay. Compounds (*S*)-**1** and (*S*,*S*)-**15** inhibited cell proliferation with minimal differences of IC_50_ values, indicating that the introduction of the second methyl at the position 5 of the side chain maintained the inhibitory activity of the parent compound (Table 7, Figure 5, and 2 Figure 2S, Supporting Information).

**Table 7:** Inhibitory Activity of Compounds (*S*)-**1** and (*S*,*S*)-**15** on Colon Cancer Cell Proliferation

IC_50_ ± SD (μM)^a^ ^,^ ^b^				
compd	SW620	SW480	HCT116	DDL-1
(*S*)-**1**	16.56 ± 0.32	5.60 ± 1.36	20.38 ± 7.39	14.13 ± 0.33
(*S*,*S*)-**15**	14.21 ± 0.40	16.40 ± 8.23	17.45 ± 5.79	15.69 ± 0.42

a The half-maximal inhibitory concentrations (IC_50_) were calculated using four-parameter logistic (4PL) symmetrical sigmoidal curves.

b Values represent the mean ± standard deviation (SD) for each cell line.

**Figure 5:**
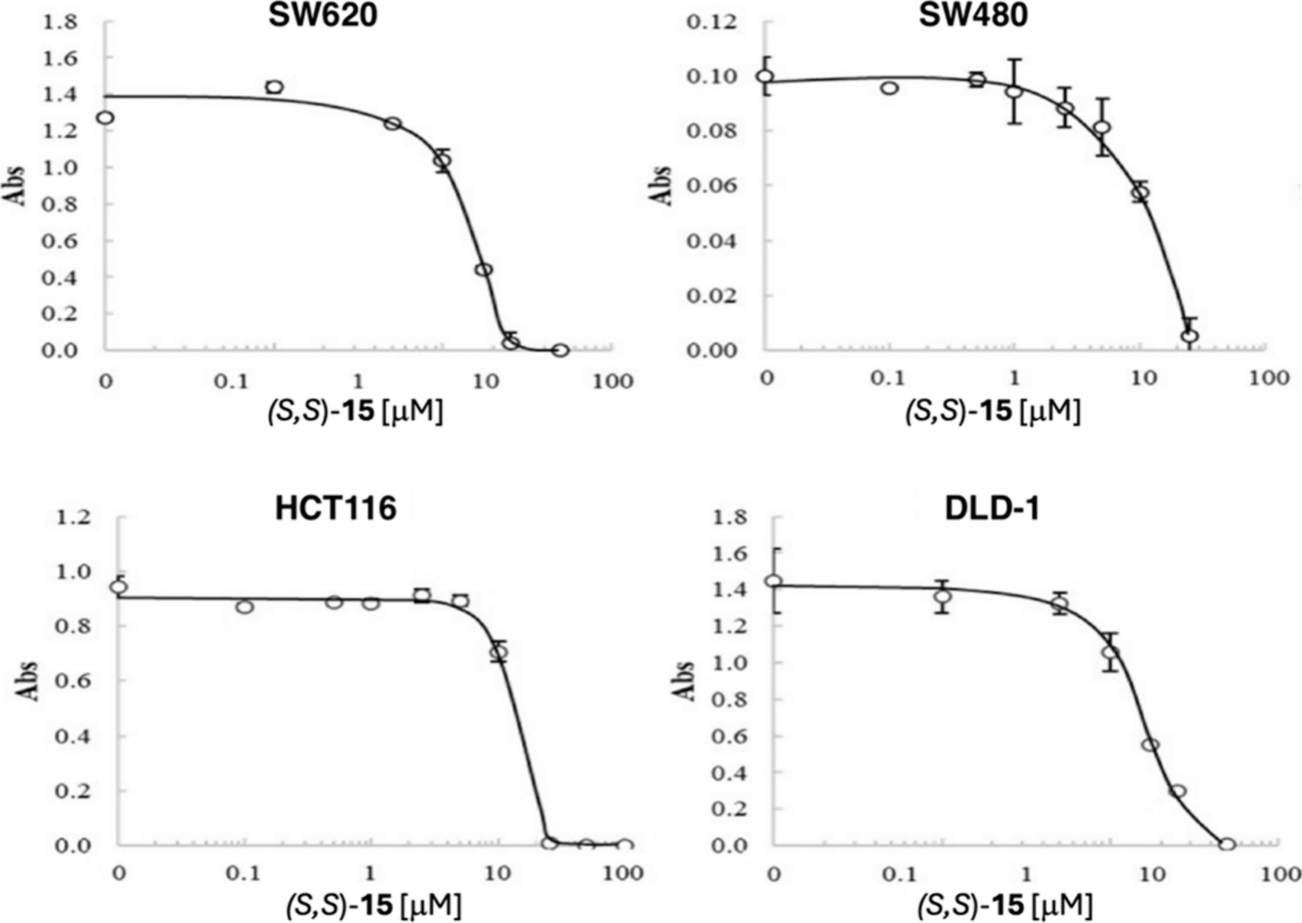
Growth inhibition of SW620, SW480, HCT116, and DLD-1 colon cancer cells treated with increasing doses of compound (*S*,*S*)-**15** (1–500 μM) or the vehicle alone (DMSO). Data are presented as mean ± SD (*n* = 4).

The induction of cell death was examined in SW620, SW480, HCT116, and DLD-1 cells treated with 50 μM (*S*)-**1** or (*S,S*)-**15** for 24 h. The presence of histone-associated DNA fragments (mono- and oligonucleosomes) in the cytoplasm, a marker of cells undergoing apoptosis, was measured by photometric enzyme immunoassay. The results showed significant increases in apoptosis across all tested cell lines following drug exposure (Figure 6).

**Figure 6:**
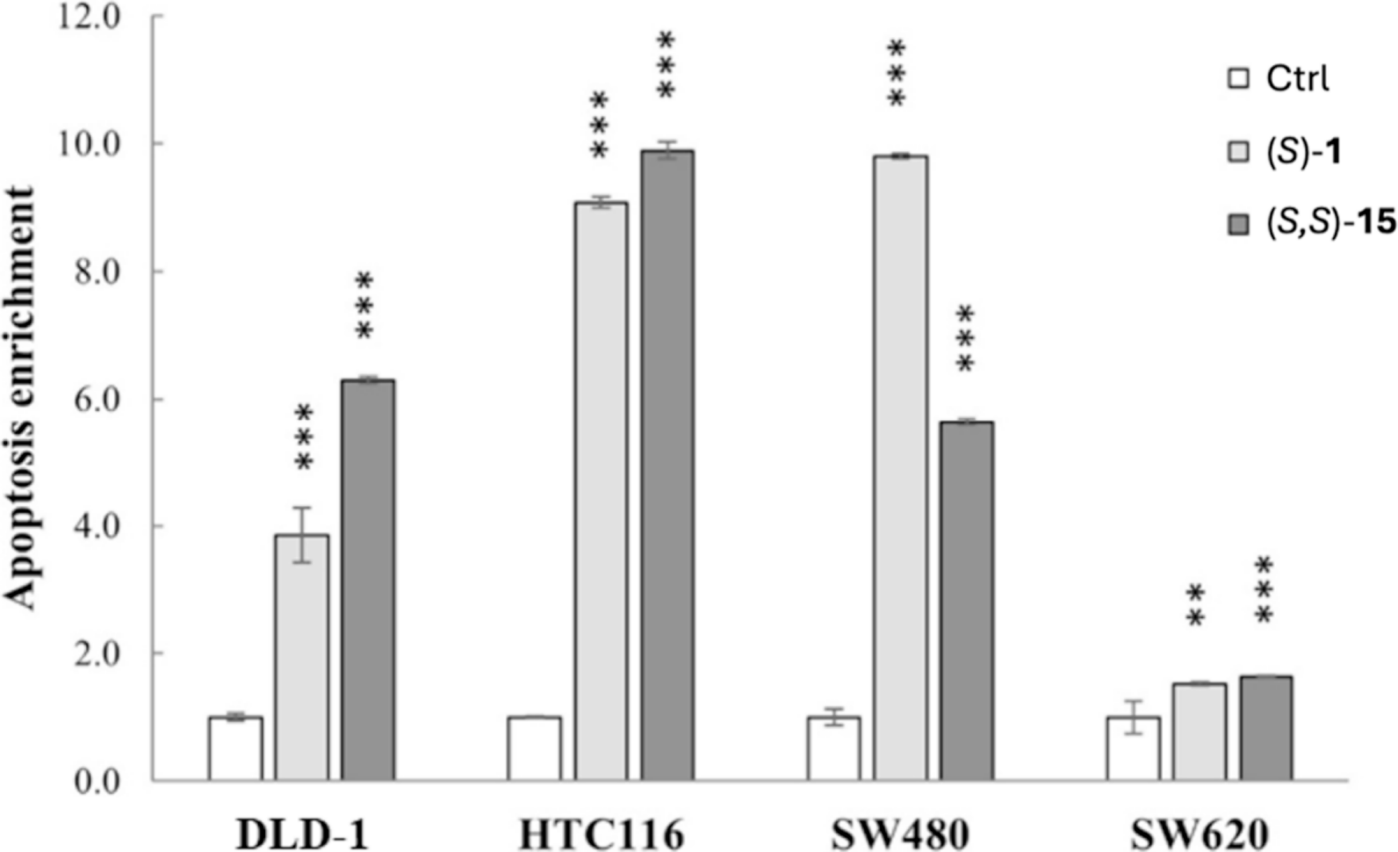
Analysis of histone-associated DNA fragments in SW620, SW480, HCT116, and DLD-1 cells treated with compound (*S*)-**1** or (*S,S*)-**15** at 50 μM, or the vehicle alone (DMSO), for 24 h. Data represent mean ± SD (*n* = 3). **p < 0.01; ***p < 0.001.

### Expression of β-Catenin in HCT116 Cells

We assessed the ability of compound (*S*,*S*)-**15** to inhibit β-catenin expression and modify its phosphorylation in the HCT116 cells. We assayed in parallel compound **67**, a β-catenin inhibitor previously developed by our research group^37^ (Figure 3S, Supporting Information) and a drug combination of (*S*,*S*)-**15** and **67**. The compounds were both used at 23 μM in single as well as in dual treatments. First, the expression of CTNNB1,^38^ the gene encoding the β-catenin protein, was evaluated by qRT-PCR experiments. The analysis revealed that, in HCT116 cells treated with either the single agents or their combination, the relative mRNA level of β-catenin was significantly lower than in DMSO-treated cells (Figure 7). In addition, the drug combination demonstrated superior efficacy compared to the individual compounds.

**Figure 7:**
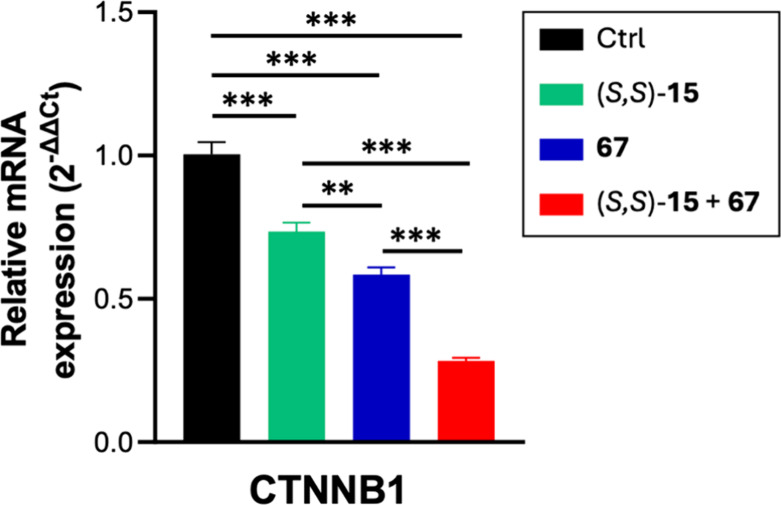
Quantitative RT-PCR analysis of CTNNB1 mRNA levels in HCT116 cells treated with DMSO (Ctrl), (*S*,*S*)-**15**, **67**, or (*S*,*S*)-**15** + **67** (23 μM each). 18S rRNA was used as a reference gene. Data represent mean ± SD (*n* = 3). **p < 0.01; ***p < 0.001.

Subsequently, the β-catenin protein level and phosphorylation status were evaluated in Western blot experiments. β-Catenin is phosphorylated at Ser675, primarily by protein kinase A (PKA);^39^ this modification promotes its interaction with the transcriptional coactivator CREB-binding protein (CBP), thereby enhancing β-catenin-mediated gene transcription. A significant reduction in total protein levels was observed across all treated HCT116 cells, with the drug combination exhibiting a more pronounced effect than compound (*S*,*S*)-**15** alone (Figure 8A,B). Compound **67** was also able to reduce the amount of phospho-Ser675 β-catenin (Figure 8C).

**Figure 8:**
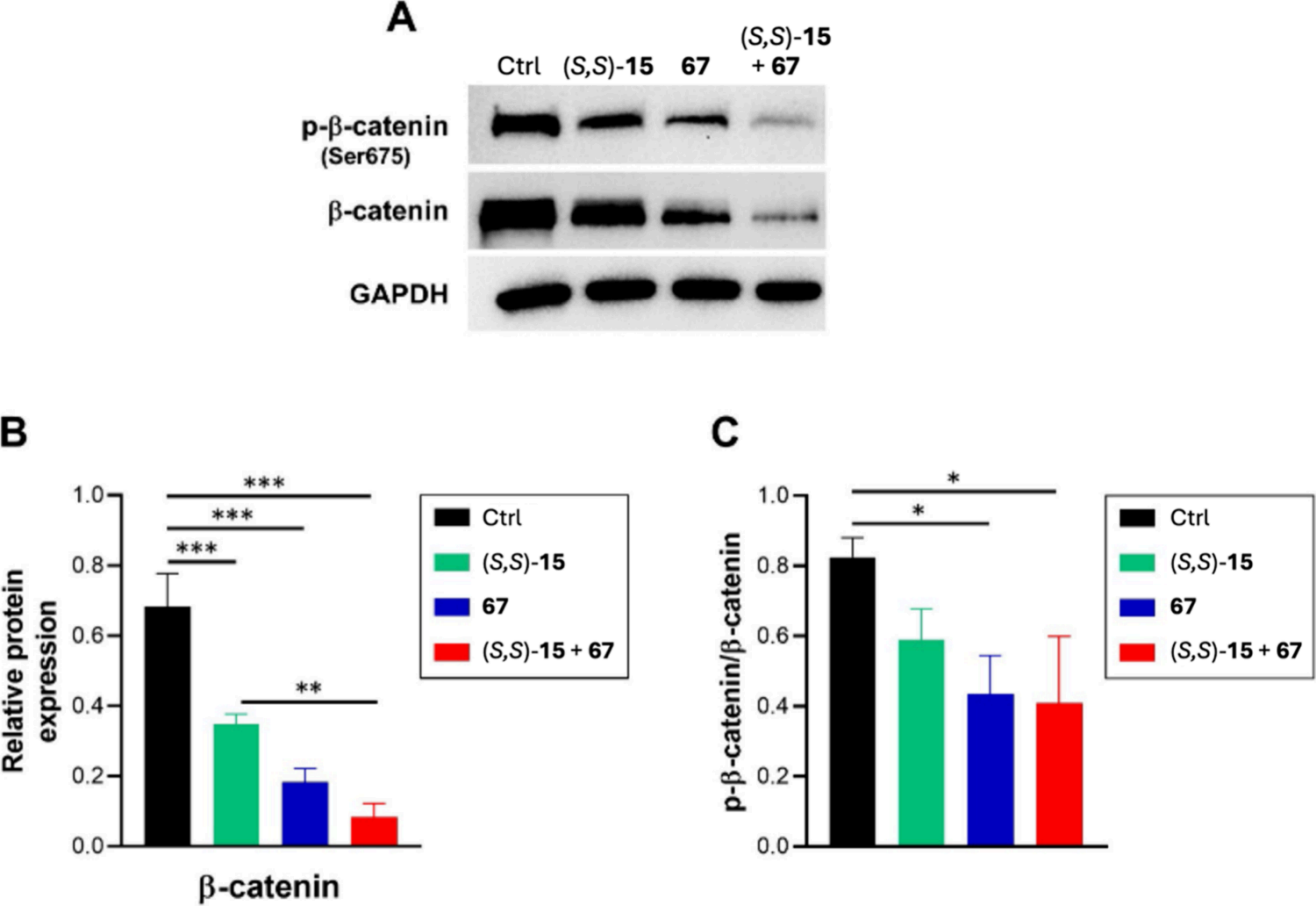
Western blot assay of β-catenin levels in HCT116 cells treated with DMSO (Ctrl), (*S*,*S*)-**15**, **67**, or (*S*,*S*)-**15** + **67** (23 μM each). (A) Representative immunoblot of total β-catenin, p-β-catenin (Ser675), and glyceraldehyde-3-phosphate dehydrogenase (GAPDH) as loading control. (B) Densitometric analysis of the total β-catenin in control and treated cells. (C) p-β-catenin/total β-catenin ratio in control and treated cells. Data represent mean ± SD (*n* = 3). *p < 0.05; **p < 0.01; ***p < 0.001.

### HCT116 Xenograft Model

5-Fluorouracil (5-FU) is a first-line drug for CRC acting through multiple mechanisms. Specifically, 5-FU blocks DNA synthesis by inhibiting thymidylate synthase, and it incorporates into DNA and RNA causing single- and double-strand breaks that lead to p53 activation and subsequent apoptosis. A recent work reported that 5-FU cytotoxicity results from interference with ribosome biogenesis, and lysosomal degradation of rRNA leading to apoptosis in both p53 WT and mutant cell lines.^40^ However, the long-term eradication of CRC is often compromised by disease recurrence and the development of chemoresistance. In recent years, various combination therapies targeting distinct oncogenic pathways are being tested to circumvent these issues. In particular, some in vitro and in vivo studies have reported synergistic anticancer effects when combining 5-FU with Wnt/β-catenin inhibitors, such as iCRT3 and loganetin.^41,42^ On this basis, we compared the efficacy of (*S*,*S*)-**15** and 5-FU in suppressing tumor growth in BALB/C^nu/nu^ mice inoculated subcutaneously with HCT116 cells in the logarithmic growth phase. Moreover, we investigated the effectiveness of a (*S,S*)-**15** and 5-FU drug combination.

After tumorigenesis, the mice were divided in four groups and received intraperitoneal injections with saline (an isotonic 0.9% sodium chloride solution) as control (Ctrl), compound (*S*,*S*)-**15** at 25 mg/kg, 5-FU at 23 mg/kg,^43^ or a drug combination of (*S*,*S*)-**15** at 14 mg/kg and 5-FU at 12 mg/kg every 2 days for 40 days. The 5-FU dosage was determined via body surface area (BSA)-based scaling for interspecies dose translation from humans to animals.^44^

After mouse euthanasia, tumors were harvested from the backs, weighed, and measured. Tissue sections were stained with hematoxylin and eosin (H&E) to assess tumor morphology (Figure 9A). The results showed that tumors from (*S*,*S*)-**15**, 5-FU, and 1:1 (*S*,*S*)-**15** and 5-FU drug combination were significantly smaller than those from the control group in terms of both tumor volume and weight (Figures 9B and Figure 4S, Supporting Information). Interestingly, compound (*S*,*S*)-**15**, either alone or combined with 5-FU, exhibited a slightly superior efficacy over 5-FU administered as a single agent (Figures 10A,B).

**Figure 9:**
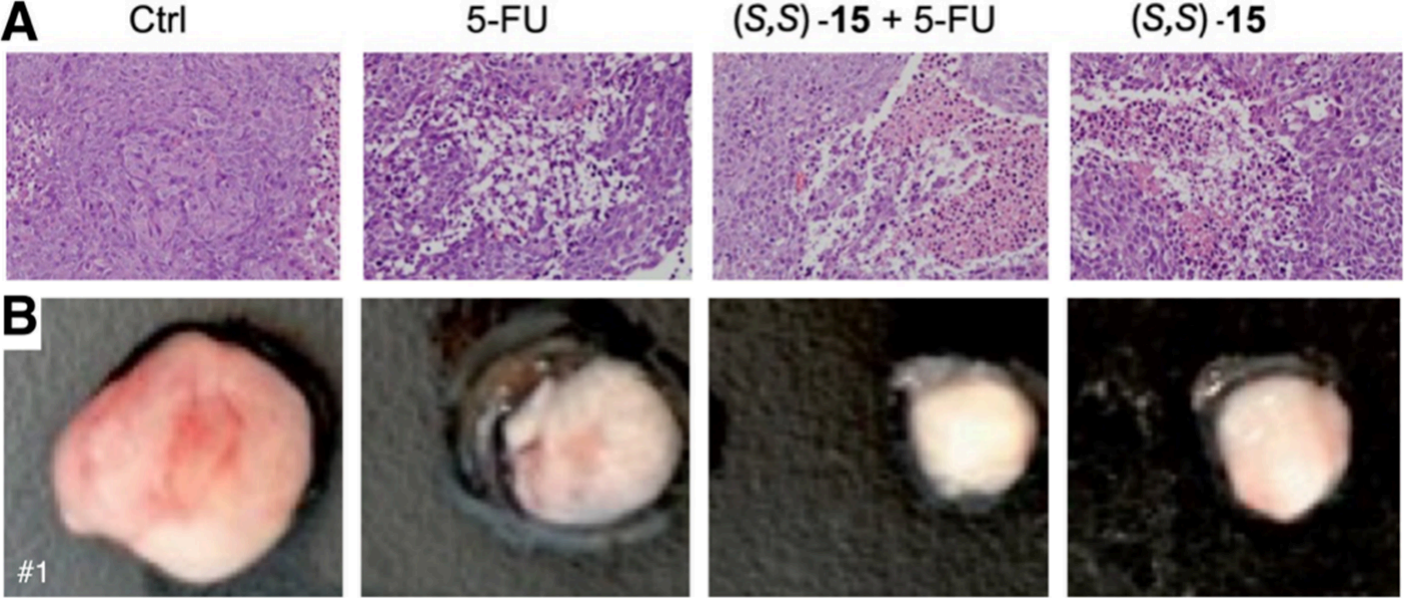
(A) H&E staining of four representative tissue sections of CRC xenografts in the different animal groups. Magnification 200×. (B) Example #1 of tumor tissues collected from the backs of the mice treated with saline, (*S*,*S*)-**15** (25 mg/kg), 5-FU (23 mg/kg), or 1:1 (*S,S*)-**15** (14 mg/kg) + 5-FU (12 mg/kg) drug combination.

**Figure 10:**
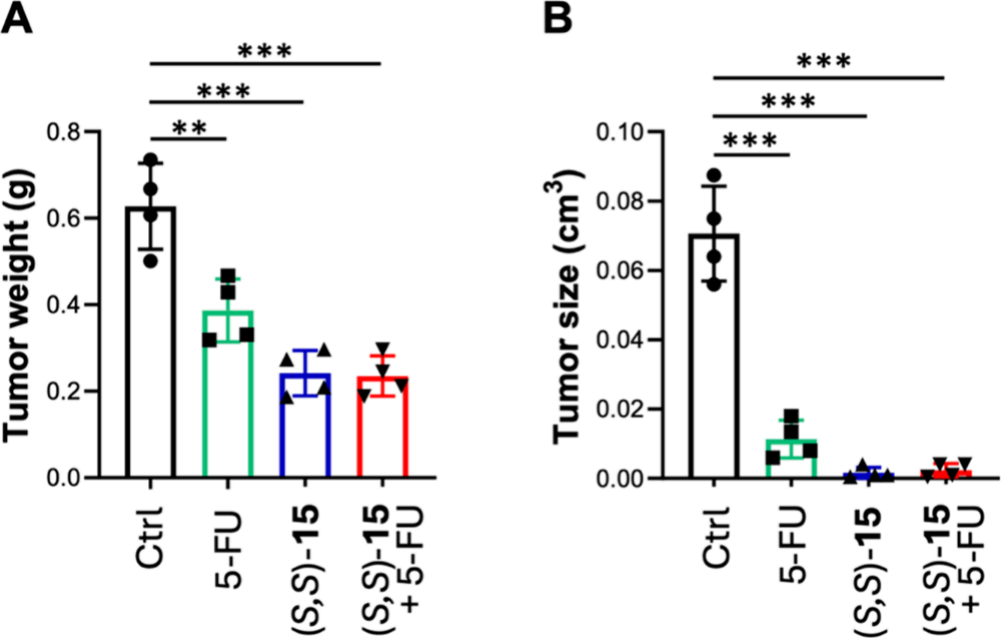
(*S*,*S*)-**15**, 5-FU and 1:1 (*S*,*S*)-**15** + 5-FU drug combination suppress in vivo tumorigenicity of the human HCT116 CRC cell line. (A) Tumor weights. (B) Tumor volumes. Data are expressed as mean ± SD (*n* = 4). **p < 0.01; ***p < 0.001 (*n* = 4).

### Expression of β-Catenin in Tumor Tissues

Xenograft tissues were examined for the expression of β-catenin at both transcriptional and translational levels. The relative CTNNB1 mRNA expression in tumor tissues treated with (*S*,*S*)-**15**, 5-FU, or a 1:1 drug combination of (*S*,*S*)-**15** with 5-FU was significantly lower than that observed in the control group (Figure 11). Moreover, compound (*S*,*S*)-**15** induced a greater mRNA reduction compared to 5-FU.

**Figure 11:**
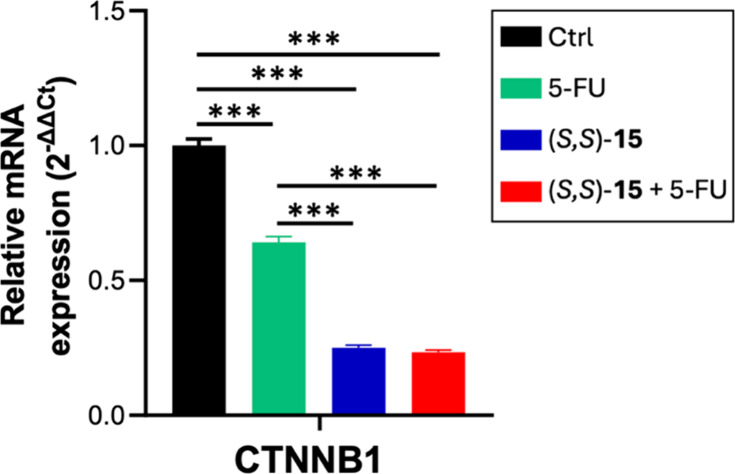
Quantitative RT-PCR analysis of CTNNB1 mRNA levels in tumor xenografts from mice treated with saline (Ctrl), (*S*,*S*)-**15**, 5-FU, or 1:1 (*S,S*)-**15** + 5-FU. Data represent mean ± SD (*n* = 4). ***p < 0.001.

These findings were mirrored in Western blot and immunofluorescence experiments, which evidenced a more relevant reduction of β-catenin protein in tumor tissues from mice treated with (*S*,*S*)-**15** (Figure 12A,B). The down-regulation of both β-catenin mRNA and protein by (*S*,*S*)-**15** is not unexpected. Indeed, while Wnt signaling primarily induces protein stabilization, β-catenin can act as a cotranscription factor for its own promoter, thus triggering a positive feedback loop.^45^

**Figure 12:**
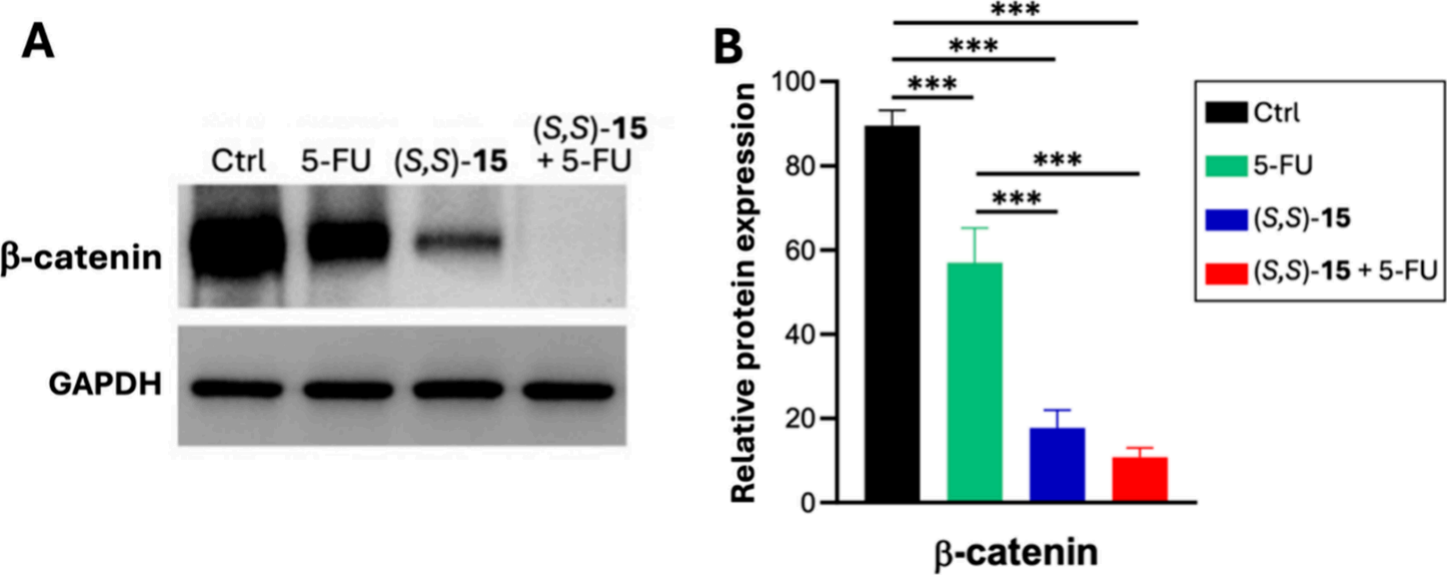
(A) Representative Western blot assay of β-catenin level in tumor xenografts from mice treated with saline, 5-FU, (*S*,*S*)-**15**, and 1:1 (*S*,*S*)-**15** + 5-FU. Glyceraldehyde-3-phosphate dehydrogenase (GAPDH) was used as a loading control. (B) Densitometric analysis of Western blot results. Data represent mean ± SD (*n* = 4). ***p < 0.001.

The human *K*_i_-67 protein is routinely used as a marker for determining the growth fraction of a cell population. *K*_i_-67 is present during all active phases of the cell cycle but is absent in resting (G_0_) cells, and its expression is an absolute requirement for progression through the cell-division cycle.^46^ Immunohistochemical results showed that, compared with tumors from the control group, tumors from the 5-FU, (*S*,*S*)-**15**, and 1:1 (*S*,*S*)-**15** + 5-FU treatment groups had a significantly reduced *K*_i_-67 expression. In particular, the tumor tissues treated with the 1:1 (*S*,*S*)-**15** + 5-FU drug combination showed the lowest *K*_i_-67 levels (Figure 13A,B).

**Figure 13:**
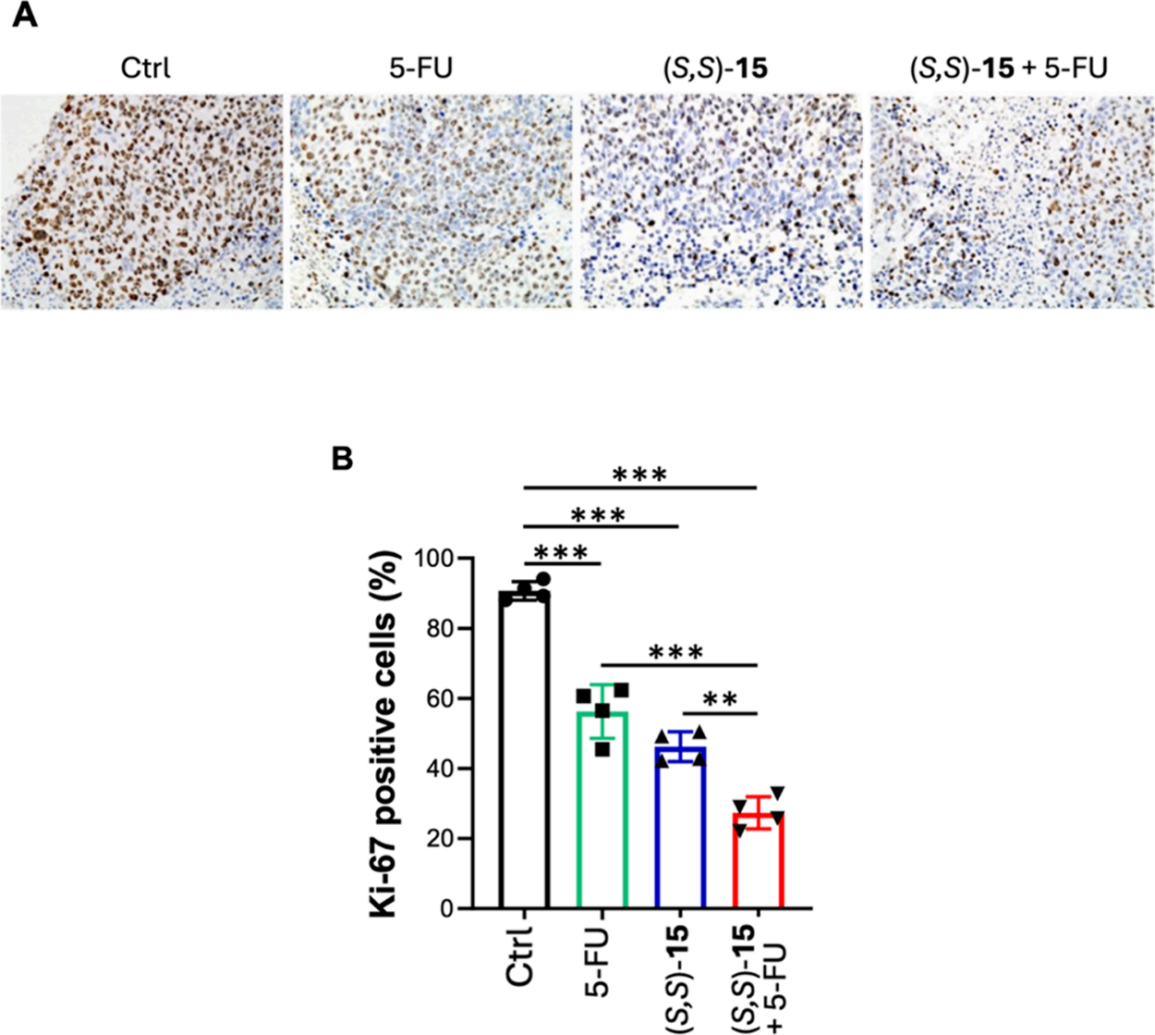
Immunohistochemical staining of *K*_i_-67 in tumor xenografts. (A) Representative images of *K*_i_-67 positive (*K*_i_-67+) cells in tumor sections from Ctrl, 5-FU, (*S*,*S*)-**15**, and 1:1 (*S*,*S*)-**15** + 5-FU treatment groups. Magnification 200×. (B) Quantification of *K*_i_-67+ cells expressed as a percentage of total tumor cells in each group (*n* = 4). Data represent mean ± SD (*n* = 4). ***p < 0.001.

### P-Glycoprotein (P-gp)

One major mechanism of MDR is linked to ABC membrane transporters. A close correlation between up-regulation of DVL1–3 and overexpressions of the P-gp and other ABC transporters in CRC HCT-8 cells was described by Zhang et al.^17^ The expressions of P-gp increased by up-regulation of DVL1–3 in the HCT-8 cells but decreased by down-regulation of DVL1–3 using an inhibitor of the PDZ domain of DVL. The data by Zhang et al. indicated that DVL positively controlled the expression of P-gp to facilitate MDR in CRC.^17^ Thus, we aimed to evaluate the efficacy of compound (*S*,*S*)-**15** to decrease the activity of the P-gp. We used the colon cancer HT29 cell line, which is doxorubicin (DOX) sensitive because it expresses low levels of P-gp, and its drug-resistant counterpart, the HT29/DX cell line. The latter cell line was selected stepwise in media with increasing concentrations of DOX resulting in expression of a high level of P-gp.^47^ The HT29 and HT29/DX pair has been extensively used and characterized by our group^48,49^ as a cancer model for DOX resistance with important translational potential. A major drawback of the HT29/DX cells is the expression of other ABC transporters, including MRP1, MRP2, MRP3, MRP5, and BCRP.^47^ Preliminarily, the cytotoxicity of compound (*S,S*)-**15** alone was assessed. Compound (*S,S*)-**15** showed dose-dependent cytotoxicity that became significant at concentrations >1 μM, with no significant differences on the HT29/HT29/DX model. Based on the lack of toxicity in the 1–100 nM range of concentrations, we used these concentrations for the next experiments (Figure 14A).

**Figure 14:**
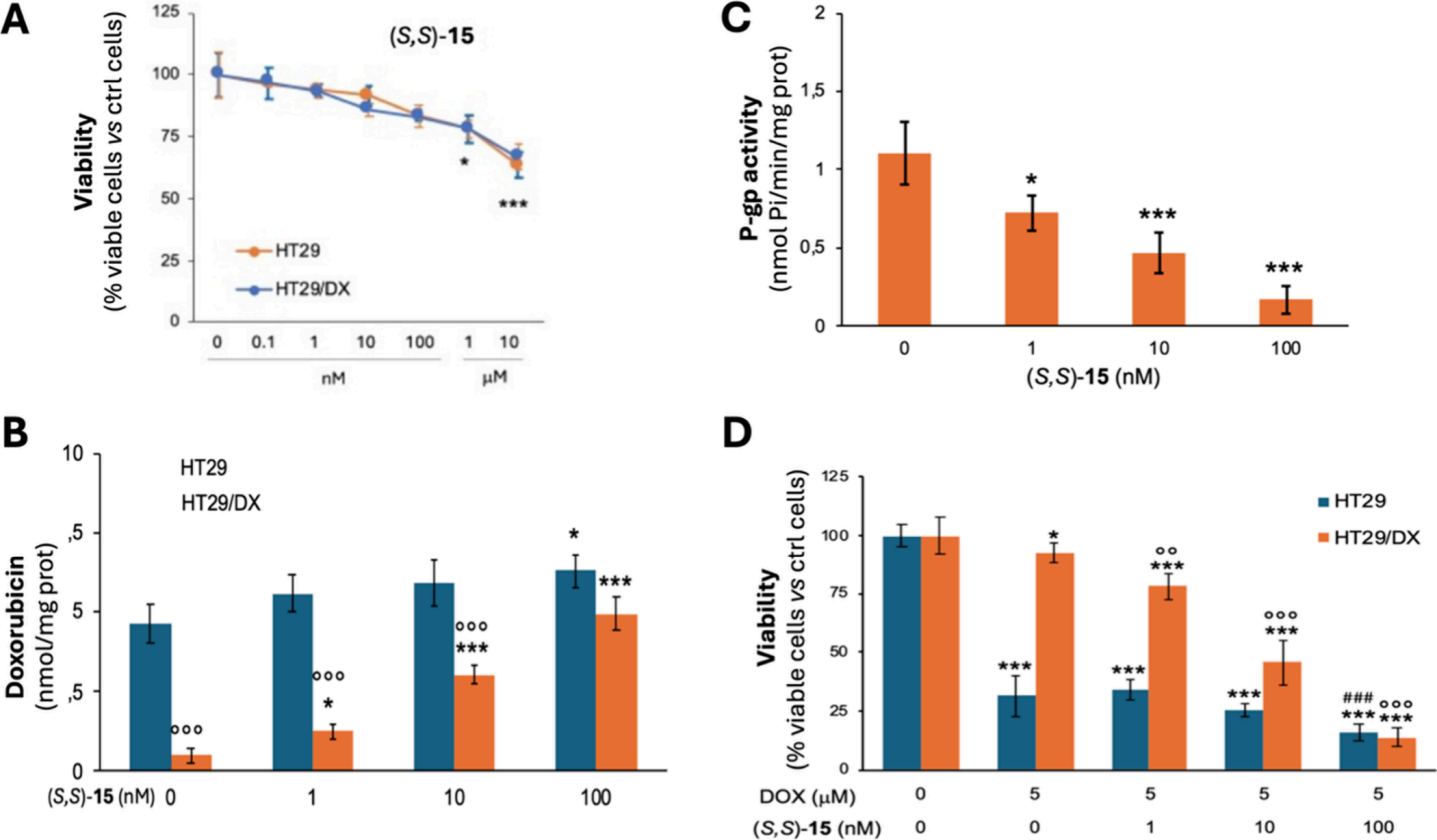
(A) Dose-dependent effects of (*S*,*S*)-**15** on the viability of HT29 and HT29/DX cells lines. Cells were incubated with increasing concentrations of (*S*,*S*)-**15**, from 0 (DMSO) to 10 μM, for 72 h, and then the viability was measured with a spectrophotometric MTT assay. Data are means ± SD (*n* = 3). *p < 0.05, ***p < 0.001: versus untreated cells. (B) Intracellular accumulation of DOX in HT29 and HT29/DX cells treated with increasing doses of (*S,S*)-**15**. Cells were incubated for 3 h with 5 μM DOX, in the absence or presence of the compound (*S*,*S*)-**15** at 1, 10, and 100 nM. The intracellular drug retention was measured fluorimetrically. Data are means ± SD (*n* = 3); *p < 0.05, ***p < 0.001 versus untreated HT29 cells; °°°*p* < 0.001 versus untreated HT29/DX cells. (C) Compound (*S*,*S*)-**15** dose-dependently inhibited the activity of P-gp extracted in nondenaturing conditions from HT29-DX cells. The protein was treated for 3 h with (*S*,*S*)-**15** at 1–100 nM concentrations, and the rate of ATP hydrolysis was measured spectrophotometrically. *p < 0.05, ***p < 0.001. (D) Viability of HT29 and HT29/DX cells incubated with DMSO (Ctrl), 5 μM DOX alone, or 5 μM DOX and (*S*,*S*)-**15** at 1, 10, or 100 nM for 72 h. Viable cells were measured by MTT assay and expressed as % relative to DMSO-treated cells. Viability of HT29 and HT29/DX cells treated with 100 nM (*S*,*S*)-**15** alone was 83.6 ± 4.3 and 83.0 ± 2.3%, respectively. Data are means ± SD (*n* = 4). *p < 0.05, ***p < 0.001: treated versus untreated cells; ###*p* < 0.001: HT29 cells treated with DOX + (*S*,*S*)-**15** versus HT29 cells treated with DOX alone; °°*p* < 0.01, °°°*p* < 0.001: HT29/DX cells treated with DOX + (*S*,*S*)-**15** versus HT29/DX cells treated with DOX alone.

The intracellular accumulation of DOX, a typical substrate of P-gp,^50^ was first evaluated. HT29 showed the expected basally higher accumulation of the drug compared with the resistant counterpart HT29/DX cells. Compound (*S,S*)-**15** did not change the intracellular amount of DOX retained within HT29 cells. On the contrary, (*S,S*)-**15** induced a strong dose-dependent increase of DOX accumulation in HT29/DX cells; notably, 100 nM (*S*,*S*)-**15** reached the same level of intracellular drug level detected in sensitive HT29 cells (Figure 14B).

We next investigated the ability of (*S*,*S*)-**15** to inhibit the catalytic cycle of P-gp. P-gp consists of two nucleotide-binding domains (NBDs) and two transmembrane domains (TMDs). Conformational changes in the TMDs convert a high-affinity drug-substrate site facing inward the cell to a low-affinity site that is outward facing, and this transition is coupled to ATP binding and hydrolysis.^51^ Since P-gp takes energy from ATP, the rate of ATP hydrolysis is considered an index of P-gp activity.^52^ The rate of ATP hydrolysis, a step necessary to efflux DOX, was measured using a P-gp extracted from HT29/DX cells in nondenaturing conditions. Compound (*S*,*S*)-**15** dose-dependently inhibited P-gp activity (Figure 14C).

The potential of compound (*S*,*S*)-**15** to reverse resistance to DOX in terms of cytotoxicity was assessed by measuring viability of cells coincubated with (*S*,*S*)-**15** and DOX at 5 μM, an already established concentration to discriminate sensitive versus resistant cells.^53^ DOX alone decreased viability of sensitive HT29 cells to about 30% but did not affect viability of resistant HT29/DX cells. In HT29 cells, only the 100 nM concentration of (*S*,*S*)-**15** significantly reduced cell viability compared with DOX alone. On the contrary, in HT29/DX cells treated with compound (*S*,*S*)-**15**, the cell viability decreased in a dose-dependent manner restoring the sensitivity to DOX. In particular, the 5 μM DOX and 100 nM (*S*,*S*)-**15** drug combination induced higher cytotoxicity in HT29/DX cells than DOX alone in the parental chemosensitive HT29 line (*p* < 0.001), confirming full overcoming of the resistance to DOX (Figure 14D).

### Pharmacokinetic (PK) Studies

To determine pharmacokinetic parameters following intravenous (IV) administration, male CD1 mice were dosed with (*S*,*S*)-**15** at 1 mg/kg using a formulation containing 5% DMSO in phosphate-buffered saline (PBS) at a concentration of 0.2 mg/mL. Blood samples were collected at 5 min, 20 min, 1 h, 2 h, 4 h, and 7 h postdose. After IV bolus administration, (*S*,*S*)-**15** displayed a moderate systemic clearance of 46 mL/min/kg, and an elimination half-life (*T*_1/2_) of approximately 102 min. For oral administration (OS), (*S*,*S*)-**15** was administered at 5 mg/kg using the same vehicle (5% DMSO in PBS) at a concentration of 0.5 mg/mL, with blood sampling at 15 min, 30 min, 1 h, 2 h, 4 h, and 7 h postdose. Oral plasma exposure was low, resulting in a very limited absolute bioavailability (0.5%), with a Tmax of 30 min. Plasma concentration–time profiles following oral dosing exhibited pronounced intrasubject variability, suggesting a solubility-limited absorption under the tested formulation conditions. Overall, these data indicate that (*S*,*S*)-**15** shows acceptable pharmacokinetic behavior following IV administration, whereas oral dosing is associated with low and variable systemic exposure.

## Conclusions

We synthesized new indole-2-carboxamides **2–18** as DVL1 inhibitors. We focused structure–activity relationship (SAR) studies on (*i*) position 5 of the indole nucleus, (*ii*) positions 3′,5′ of the phenyl ring, (*iii*) bridging group, (*iv*) position 5 of the side chain, and (*v*) the terminal pyridinyl group. Introduction of a methyl group at C5 of the side chain of the carboxamide nitrogen was predicted by the SwissADME and SMARTCyp servers to benefit the drug-like properties, compared to compound (*S*)-**1**. We separated the four enantiomers of **15** by enantioselective HPLC. Compound (*S*,*S*)-**15** showed the most potent DVL1 inhibition with an IC_50_ value of 0.97 ± 0.21 μM, and at 10 μM completely abolished the binding between the DVL1 domain PDZ and TMEM88 demonstrating specific interaction with the DVL1 PDZ. Compound (*S*,*S*)-**15** was also obtained by enantioselective synthesis by coupling reaction that did not affect the stereogenic centers. Compound (*S*,*S*)-**15** demonstrated superior metabolic stability compared to the reference (*S*)-**1** in human liver microsomes and significant metabolic stability during the IV administration in PK studies. Treatment of HCT116 CRC cells with (*S*,*S*)-**15** decreased β-catenin mRNA and protein expression. The dose-dependent anticancer effect of (*S*,*S*)-**15** was demonstrated *in vitro* across various CRC human cell lines, resulting in growth arrest and apoptosis induction.

In the xenograft model, (*S*,*S*)-**15** reduced significantly both volume and weight of tumor masses. The combination of (*S*,*S*)-**15** + 5-FU was no more effective than (*S*,*S*)-**15** alone. However, this drug combination caused stronger reduction of β-catenin levels and of the proliferation marker *K*_i_-67, compared to either 5-FU or (*S*,*S*)-**15** alone. According to the described correlation between up-regulation of DVL1–3 and overexpressions of the P-glycoprotein (P-gp) in CRC HCT-8 cells,^17^ compound (*S*,*S*)-**15** inhibited the P-gp producing the same decrease in cell viability achieved by DOX alone in the HT29 chemosensitive cells.

In summary, our results indicate that compound (*S*,*S*)-**15** is a novel dual targeting agent by specific binding to the PDZ domain of the DVL1 protein of the Wnt/β-catenin pathway and inhibition of the P-gp. Compound (*S*,*S*)-**15** lowered the expression levels of β-catenin and inhibited the CRC cell growth *in vitro* as well as in a xenograft model. Furthermore, (*S*,*S*)-**15** exhibited improved metabolic stability compared to the reference compound (*S*)-**1**, particularly in human liver microsomes, and acceptable pharmacokinetic properties following intravenous administration, indicating that metabolic clearance is not a major limitation for this chemotype. Oral administration, however, resulted in low and variable systemic exposure, consistent with absorption-related constraints. From a pharmacodynamic perspective, (*S,S*)-**15** represents a valuable tool compound for supporting *in vivo* proof-of-concept studies using nonoral routes of administration. Collectively, these findings provide insight into the pharmacokinetic behavior of this compound class and prompt future efforts aimed at improving oral exposure through optimization of compound properties, formulation strategies, and evaluation of alternative routes of administration.

## Experimental Section

### Chemistry

#### Synthesis

All reagents and solvents were handled according to the material safety data sheet of the suppliers and used as purchased without further purification. Organic solutions were dried over anhydrous sodium sulfate. Evaporation of solvents was carried out on a Büchi Rotavapor R-210 equipped with a Büchi V-850 vacuum controller and a Büchi V-700 vacuum pump. Column chromatography was performed on columns packed with silica gel from Macherey–Nagel (70–230 mesh). Silica gel thin-layer chromatography (TLC) cards from Macherey-Nagel (silica gel-precoated aluminum cards with a fluorescent indicator visualizable at 254 nm) were used for TLC. Developed plates were visualized with a Spectroline ENF 260C/FE UV apparatus. Melting points (mp) were determined on a Stuart Scientific SMP1 apparatus and are uncorrected. Infrared (IR) spectra were recorded on a PerkinElmer Spectrum 100 FT-IR spectrophotometer equipped with a universal attenuated total reflectance accessory, and IR data were acquired and processed by PerkinElmer Spectrum 10.03.00.0069 software. Band position and absorption ranges are given in cm^–1^. Proton nuclear magnetic resonance (^1^H NMR) spectra were recorded with a Bruker Avance (400 MHz) spectrometer in the indicated solvent, and the corresponding fid files were processed with MestreLab Research SL MestreReNova 6.2.1–769 software. ^1^H NMR used abbreviations: s = singlet, d = doublet, dd, double doublet, t = triplet, q = quartet, qn, quintet, tt = triplet of triplets, m = multiplet. Carbon-13 nuclear magnetic resonance (^13^C NMR) spectra were recorded with a Bruker AVANCE (100 MHz) spectrometer in the indicated solvent, and the corresponding FID files were processed by MestreLab Research SL MestreReNova 6.2.1–769 software. Chemical shifts of ^1^H and ^13^C NMR are expressed in δ units (ppm) from tetramethylsilane (δ = 0). High-resolution mass spectrometry (HRMS) analyses were performed using a microTOF-QIII (Bruker Daltonik, Germany) mass spectrometer equipped with electrospray ionization (ESI) in positive mode, capillary 4000 V, end plate offset −500 V, source temperature 180 °C, dry gas: N_2_ at 200 °C/4 L/min, nebulizer N2, 1 bar, *m*/*z* 80–3000, collision RF 300 Vpp, calibration at low concentration. Tuning Mix ESI/APCI (Agilent Technologies). Both monoisotopic/100% calculated and found values are reported. Ionization technique ESI+.

Compound purity >95% was assessed by HPLC (high-performance liquid chromatography). The HPLC system used (Dionex UltiMate 3000, Thermo Fisher Scientific Inc.) consisted of SR-3000 solvent rack, LPG-3400SD quaternary analytical pump, TCC-3000SD column compartment, DAD-3000 diode array detector, and analytical manual injection valve with a 20 μL loop. The sample was dissolved in acetonitrile (1 mg/mL). HPLC analysis was performed using an Acclaim 120 C18 column (5 mm, 4.6 mm × 250 mm, Thermo Fisher Scientific Inc.) at 25 ± 1 °C. The mobile phase consisted of water +0.1% trifluoroacetic acid and acetonitrile +0.1% trifluoroacetic acid, delivered at a flow rate of 1.0 mL/min, UV detection was performed at 206, 230, 254, and 365 nm. Chromatographic data were acquired and processed by Chromeleon 6.80 SR15b Build 4981 software (Thermo Fisher Scientific Inc.).

### General Procedure A. Preparation of Compounds **2**–**11** and **15**–**20**

#### Example. 5-Bromo-3-((3,5-dimethylphenyl)­sulfonyl)-*N*-(1-oxo-1-((pyridin-4-ylmethyl)­amino)­propan-2-yl)-1*H*-indole-2-carboxamide (**8**)

A mixture of 5-bromo-3-((3,5-dimethylphenyl)­sulfonyl)-1*H*-indole-2-carbonyl)­alanine (**26**) (0.050 g; 0.1 mmol), pyridin-4-ylmethanamine (0.014 g; 0.13 mmol), DIPEA (0.011 g; 0.11 mmol), and HATU (0.057 g; 0.11 mmol) with DMF (5 mL) was stirred under argon stream overnight at 25 °C. Then, the solution was diluted with water and extracted with ethyl acetate. The organic layer was washed with brine, dried over anhydrous sodium sulfate, and filtered. Evaporation of the solvent gave a residue that was purified with flash chromatography (DCM:EtOH = 9.9:0.1) yielding product **8** (0.030 g; 50%) as white solid, mp 171–174 °C (from ethanol). ^1^H NMR (400 MHz, DMSO-*d*_6_) δ 13.33 (br s, disappeared after treatment with D_2_O, 1H), 9.70 (br d, *J* = 6.8 Hz, disappeared after treatment with D_2_O, 1H), 8.96 (br t, *J* = 5.9 and 12.0 Hz, disappeared after treatment with D_2_O, 1H), 8.70 (d, *J* = 5.0 Hz, 2H), 8.38 (s, 1H), 7.91 (s, 2H), 7.72 (q, *J* = 9.6 Hz, 2H), 7.52 (d, *J* = 5.0 Hz, 2H), 7.48 (s, 1H), 4.86 (qn, *J* = 6.6 Hz, 1H), 4.62 (t, *J* = 4.1 Hz, 2H), 3.25–3.21 (m, 3H), 1.95 (t, *J* = 5.9 Hz, 3H), 1.68 ppm (d, *J* = 7.0 Hz, 3H). ^13^C NMR (100 MHz, DMSO-*d*_6_) δ 172.23, 159.02, 149.97, 148.73, 142.72, 148.73, 142.72, 139.61, 135.92, 135.38, 133.52, 128.04, 126.48, 124.13, 122.61, 122.41, 115.99, 115.87, 112.15, 21.14, 18.67 ppm. IR: ν 1640 and 3183 cm^–1^. HRMS (ESI^+^), *m*/*z* calculated for C_26_H_25_BrN_4_O_4_S [M + H]^+^ 569.0853 (monoisotopic), 571.0835 (100%), found 569.0853, 571.0838.

#### 5-Chloro-3-((3,5-dimethylphenyl)­sulfonyl)-*N*-(1-oxo-1-((pyridin-4-ylmethyl)­amino)­propan-2-yl)-1*H*-indole-2-carboxamide (**1**)

Resynthesized as **8** starting from **21**. Yield 60%, mp 112–114 °C. (Lit. (23) Yield 49%, mp 112–115 °C). ^1^H NMR (400 MHz, DMSO-*d*_6_) δ 13.15 (s, 1H), 9.55 (d, *J* = 6.9 Hz, 1H), 8.79 (t, *J* = 6.0 Hz, 1H), 8.56–8.53 (m, 2H), 8.08 (d, *J* = 2.1 Hz, 1H), 7.77–7.76 (m, 2H), 7.64 (d, *J* = 8.8 Hz, 1H), 7.44 (dd, *J* = 8.8 and 2.1 Hz, 1H), 7.39–7.34 (m, 2H), 7.32 (s, 1H), 4.71 (q, *J* = 7.0 Hz, 1H), 4.48–4.45 (m, 2H), 2.34 (s, 6H), 1.53 (d, *J* = 7.0 Hz, 3H) ppm. ^13^C NMR (100 MHz, DMSO-*d*_6_) δ 172.24, 159.01, 149.96, 148.73, 142.72, 139.62, 136.04, 135.38, 133.28, 127.96, 125.91, 125.54, 124.13, 122.41, 119.60, 115.55, 112.33, 49.86, 41.62, 21.13, 18.68 ppm. HRMS (ESI^+^), *m*/*z* calculated for C_26_H_25_ClN_4_O_4_S [M + H]^+^ 525.1358, found 525.1354.

#### 3-Benzoyl-5-chloro-*N*-(1-oxo-1-((pyridin-4-ylmethyl)­amino)­propan-2-yl)-1*H*-indole-2-carboxamide (**2**)

Synthesized as **8** starting from **25**. Yield 58% as white solid, mp >250 °C (from ethanol). ^1^H NMR (400 MHz, DMSO-*d*_6_) δ 12.89 (br s, disappeared after treatment with D_2_O, 1H), 9.64 (br d, *J* = 6.8 Hz, disappeared after treatment with D_2_O, 1H), 8.60 (br t, *J* = 6.0 Hz, disappeared after treatment with D_2_O, 1H), 8.47 (d, *J* = 5.0 Hz, 2H), 7.69–7.64 (m, 3H), 7.58 (d, *J* = 8.7 Hz, 1H), 7.52 (t, *J* = 7.6 Hz, 2H), 7.30 (dd, *J* = 2.0 and 8.7 Hz, 1H), 7.23 (d, *J* = 5.0 Hz, 2H), 7.17 (d, *J* = 2.0 Hz, 1H), 4.33–4.26 (m, 3H), 1.16 (d, *J* = 7.0 Hz, 3H) ppm. ^13^C NMR (100 MHz, DMSO-*d*_6_) δ 192.79, 172.40, 160.18, 149.94, 148.83, 140.52, 138.11, 133.81, 132.90, 129.41, 128.87, 128.51, 127.03, 124.88, 122.37, 120.99, 115.14, 113.53, 49.71, 41.52, 18.32. IR: ν 1634 and 3291 cm^–1^ ppm. HRMS (ESI^+^), *m*/*z* calculated for C_25_H_21_ClN_4_O_3_ [M + H]^+^ 461.1375, found 461.1373.

#### 5-Chloro-3-(3,5-dichlorobenzoyl)-*N*-(1-oxo-1-((pyridin-4-ylmethyl)­amino)­propan-2-yl)-1*H*-indole-2-carboxamide (**3**)

Synthesized as **8** starting from **24**. Yield 63% as white solid, mp >250 °C (from ethanol). ^1^H NMR (400 MHz, DMSO-*d*_6_) δ 11.87 (br s, disappeared after treatment with D_2_O, 1H), 8.77 (br d, *J* = 7.2 Hz, disappeared after treatment with D_2_O, 1H), 8.67 (br t, *J* = 6.0 Hz, disappeared after treatment with D_2_O, 1H), 8.47 (d, *J* = 5.0 Hz, 2H), 7.69–7.64 (m, 3H), 7.58 (d, *J* = 8.7 Hz, 1H), 7.52 (t, *J* = 7.6 Hz, 2H), 7.30 (dd, *J* = 2.0 and 8.7 Hz, 1H), 7.23 (d, *J* = 5.0 Hz, 2H), 7.17 (d, *J* = 2.0 Hz, 1H), 4.33–4.26 (m, 3H), 1.16 (d, *J* = 7.0 Hz, 3H) ppm. ^13^C NMR (100 MHz, DMSO-*d*_6_) δ 173.19, 161.14, 149.89, 149.08, 135.31, 133.32, 128.56, 124.67, 123.95, 122.38, 121.10, 114.35, 103.51, 49.39, 41.58, 18.34, 14.56 ppm. IR: ν 1622 and 3288 cm^–1^. HRMS (ESI^+^), *m*/*z* calculated for C_25_H_19_Cl_3_N_4_O_3_ [M + H]^+^ 529.0595, found 529.0590.

#### 5-Chloro-3-(3,5-dimethylbenzoyl)-*N*-(1-oxo-1-((pyridin-4-ylmethyl)­amino)­propan-2-yl)-1*H*-indole-2-carboxamide (**4**)

Synthesized as **8** starting from **22**. Yield 53% as pale-gray solid, mp >250 °C (from ethanol). ^1^H NMR (400 MHz, DMSO-*d*_6_) δ 12.19 (br s, disappeared after treatment with D_2_O, 1H), 9.61 (br d, *J* = 6.7 Hz, disappeared after treatment with D_2_O, 1H), 8.61 (br t, *J* = 6.0 Hz, disappeared after treatment with D_2_O, 1H), 8.48 (d, *J* = 5.0 Hz, 2H), 7.58 (d, *J* = 8.8 Hz, 1H), 7.32–7.30 (m, 4H), 7.25–7.22 (m, 3H), 4.32–4.26 (m, 3H), 2.31 (s, 6H), 1.19 (d, *J* = 7.3 Hz, 3H) ppm. ^13^C NMR (100 MHz, DMSO-*d*_6_) δ 196.31, 172.03, 160.77, 149.83, 147.60, 141.56, 138.44, 136.71, 134.90, 134.83, 129.44, 126.82, 122.40, 121.89, 121.72, 120.90, 119.84, 114.35, 43.66, 54.01, 21.63, 17.90 ppm. IR: ν 1643 and 3209 cm^–1^. HRMS (ESI^+^), *m*/*z* calculated for C_27_H_25_ClN_4_O_3_ [M + H]^+^ 489.1688, found 489.1691.

#### 5-Chloro-*N*-(1-oxo-1-((pyridin-3-ylmethyl)­amino)­propan-2-yl)-3-(phenylsulfonyl)-1*H*-indole-2-carboxamide (**5**)

Synthesized as **8** starting from **66**. Yield 50% as white solid, mp 195–198 °C (from ethanol). ^1^H NMR (400 MHz, DMSO-*d*_6_) δ 13.09 (br s, disappeared after treatment with D_2_O, 1H), 9.42 (br d, *J* = 6.9 Hz, disappeared after treatment with D_2_O, 1H), 8.67 (br t, *J* = 6.1 Hz, disappeared after treatment with D_2_O, 1H), 8.55 (dd, *J* = 1.9 and 4.4 Hz, 2H), 7.78 (d, *J* = 2.0 Hz, 1H), 7.50 (d, *J* = 8.8 Hz, 1H), 7.33 (d, *J* = 4.8 Hz, 3H), 7.25 (dd, *J* = 2.1 and 8.8 Hz, 1H), 4.59 (qn, *J* = 7.2 Hz, 1H), 4.44–4.33 (m, 2H), 4.09 (q, *J* = 7.1 Hz, 1H), 2.05 (s, 1H), 1.47 (d, *J* = 7.2 Hz, 3H) ppm. ^13^C NMR (100 MHz, DMSO-*d*_6_) δ 172.23, 159.28, 149.98, 148.72, 142.86, 136.67, 133.96, 133.34, 129.97, 127.98, 126.81, 125.75, 125.48, 122.42, 119.42, 115.57, 112.04, 49.97, 41.61, 18.53 ppm. IR: ν 1626 and 3280 cm^–1^. HRMS (ESI^+^), *m*/*z* calculated for C_24_H_21_ClN_4_O_4_S [M + H]^+^ 497.1045, found 497.1048.

#### 5-Chloro-3-((3,5-dichlorophenyl)­sulfonyl)-*N*-(1-oxo-1-((pyridin-4-ylmethyl)­amino)­propan-2-yl)-1*H*-indole-2-carboxamide (**6**)

Synthesized as **8** starting from **50**. Yield 65%, as white solid, mp 222–225 °C (from ethanol). ^1^H NMR (400 MHz, DMSO-*d*_6_) δ 13.22 (br s, disappeared after treatment with D_2_O, 1H), 9.36 (br s, disappeared after treatment with D_2_O, 1H), 8.64 (br t, *J* = 6.1 Hz, disappeared after treatment with D_2_O, 1H), 8.49 (dd, *J* = 1.6 and 4.4 Hz, 2H), 8.15 (d, *J* = 1.8 Hz, 2H), 8.01 (d, *J* = 1.8 Hz, 1H), 7.95 (t, *J* = 1.9 Hz, 1H), 7.59 (d, *J* = 8.8 Hz, 1H), 7.37 (dd, *J* = 1.8 and 9.0 Hz, 1H), 7.30 (dd, *J* = 1.6 and 4.4 Hz, 2H), 4.61 (qn, *J* = 6.9 Hz, 1H), 4.45–4.34 (m, 2H), 1.43 (d, J = 7.1 Hz, 3H) ppm. ^13^C NMR (100 MHz, DMSO-*d*_6_) δ 172.19, 159.68, 149.94, 148.71, 146.07, 138.45, 135.60, 133.47, 128.25, 125.73, 125.48, 122.41, 119.20, 115.64, 110.59, 49.86, 41.60, 18.49 ppm. IR: ν 1643 and 3262 cm^–1^. HRMS (ESI^+^), *m*/*z* calculated for C_24_H_19_Cl_3_N_4_O_4_S [M + H]^+^ 565.0265, found 565.0259.

#### 3-((3,5-Dimethylphenyl)­sulfonyl)-5-fluoro-*N*-(1-oxo-1-((pyridin-4-ylmethyl)­amino)­propan-2-yl)-1*H*-indole-2-carboxamide (**7**)

Synthesized as **8** starting from **60**. Yield 90% as white solid, mp 187–190 °C (from ethanol). ^1^H NMR (400 MHz, DMSO-*d*_6_) δ 12.93 (br s, disappeared after treatment with D_2_O, 1H), 9.39 (br s, disappeared after treatment with D_2_O, 1H), 8.65 (br t, *J* = 7.2 Hz, disappeared after treatment with D_2_O, 1H), 8.42 (dd, *J* = 1.5 and 4.0 Hz, 2H), 7.67 (dd, *J* = 2.4 and 9.8 Hz, 1H), 7.64 (s, 2H), 7.50 (quart, *J* = 4.9 Hz, 1H), 7.23 (dd, *J* = 1.5 and 4.2 Hz, 2H), 7.15 (s, 2H), 4.57 (qn, *J* = 7.3 Hz, 1H), 4.33 (m, 2H), 2.21 (s, 6H), 1.39 (d, *J* = 7.0 Hz, 3H) ppm. ^13^C NMR (100 MHz, DMSO-*d*_6_) δ 172.29, 150.06, 149.97, 148.73, 142.82, 139.58, 135.32, 125.56, 124.13, 122.41, 115.54, 114.29, 114.02, 105.52, 105.26, 49.83, 41.62, 21.12, 18.70 ppm. IR: ν 1645 and 3267 cm^–1^. HRMS (ESI^+^), *m*/*z* calculated for C_26_H_25_FN_4_O_4_S [M + H]^+^ 509.1653, found 509.1654.

#### 3-((3,5-Dimethylphenyl)­sulfonyl)-5-methyl-*N*-(1-oxo-1-((pyridin-4-ylmethyl)­amino)­propan-2-yl)-1*H*-indole-2-carboxamide (**9**)

Synthesized as **8** starting from **55**. Yield 98% as white solid, mp 161–164 °C (from ethanol). ^1^H NMR (400 MHz, DMSO-*d*_6_) δ 12.78 (br s, disappeared after treatment with D_2_O, 1H), 9.55 (br d, *J* = 7.2 Hz, disappeared after treatment with D_2_O, 1H), 8.74 (br t, *J* = 6.3 Hz, disappeared after treatment with D_2_O, 1H), 8.50 (dd, *J* = 1.7 and 4.8 Hz, 2H), 7.86 (s, 1H), 7.70 (d, *J* = 1.5 Hz, 2H), 7.45 (d, *J* = 8.3 Hz, 1H), 7.31 (dd, *J* = 1.5 and 4.6 Hz, 2H), 7.22–7.12 (m, 2H), 4.66 (qn, *J* = 7.4 Hz, 1H), 4.42–4.40 (m, 2H), 2.44 (s, 3H), 2.27 (m, 6H), 1.48 (s, 3H) ppm. ^13^C NMR (100 MHz, DMSO-*d*_6_) δ 172.37, 159.31, 149.96, 148.77, 143.10, 139.48, 135.15, 134.18, 133.10, 132.48, 127.05, 125.33, 125.21, 124.02, 122.41, 119.96, 113.38, 112.00, 55.39, 49.82, 41.62, 18.72 ppm. IR: ν 1640 and 3284 cm^–1^. HRMS (ESI^+^), *m*/*z* calculated for C_27_H_28_ClN_4_O_4_S [M + H]^+^ 505.1904, found 505.1908.

#### 5-Chloro-3-((3,5-dimethylphenyl)­sulfonyl)-*N*-(1-oxo-1-((pyridin-2-ylmethyl)­amino)­propan-2-yl)-1*H*-indole-2-carboxamide (**10**)

Synthesized as **8** starting from **21**. Yield 76% as white solid, mp 216–219 °C (from ethanol). ^1^H NMR (400 MHz, DMSO-*d*_6_) δ 13.05 (br s, disappeared after treatment with D_2_O, 1H), 9.43 (br d, *J* = 7.0 Hz, disappeared after treatment with D_2_O, 1H), 8.72 (br t, *J* = 6.0 Hz, disappeared after treatment with D_2_O, 1H), 8.51 (dd, *J* = 1.6 and 4.7 Hz, 1H), 8.03 (d, *J* = 2.0 Hz, 1H), 7.74 (td, *J* = 1.8 and 7.7 Hz, 1H), 7.69 (s, 2H), 7.57 (d, *J* = 8.8 Hz, 1H), 7.38–7.34 (m, 2H), 7.27 (t, *J* = 6.6 Hz, 2H), 4.67 (qn, *J* = 7.0 Hz, 1H), 4.46 (d, *J* = 6.9 Hz, 2H), 2.29 (s, 6H), 1.45 (d, *J* = 7.0 Hz, 3H) ppm. ^13^C NMR (100 MHz, DMSO-*d*_6_) δ 172.11, 158.91, 158.86, 149.33, 142.75, 139.61, 137.21, 136.03, 135.37, 133.28, 127.95, 125.97, 125.52, 124.14, 122.61, 121.23, 119.63, 115.56, 112.35, 49.82, 44.71, 21.14, 18.80 ppm. IR: ν 1644 and 3214 cm^–1^. HRMS (ESI^+^), *m*/*z* calculated for C_26_H_25_ClN_4_O_4_S [M + H]^+^ 525.1357, found 525.1358.

#### 5-Chloro-3-((3,5-dimethylphenyl)­sulfonyl)-*N*-(1-oxo-1-((pyridin-3-ylmethyl)­amino)­propan-2-yl)-1*H*-indole-2-carboxamide (**11**)

Synthesized as **8** starting from **21**. Yield 50% as white solid, mp >250 °C (from ethanol). ^1^H NMR (400 MHz, DMSO-*d*_6_) δ 13.05 (br s, disappeared after treatment with D_2_O, 1H), 9.43 (br d, *J* = 7.0 Hz, disappeared after treatment with D_2_O, 1H), 8.67 (br t, *J* = 6.0 Hz, disappeared after treatment with D_2_O, 1H), 8.53 (d, *J* = 2.2 Hz, 1H), 8.46 (dd, *J* = 1.6 and 4.7 Hz, 1H), 8.02 (d, *J* = 2.0 Hz, 1H), 7.71–7.68 (m, 3H), 7.57 (d, *J* = 8.8 Hz, 1H), 7.38–7.32 (m, 2H), 7.27 (s, 1H), 4.61 (qn, *J* = 7.0 Hz, 1H), 4.45–4.35 (m, 2H), 2.29 (s, 6H), 1.43 (d, *J* = 7.0 Hz, 3H) ppm. ^13^C NMR (100 MHz, DMSO-*d*_6_) δ 172.06 158.96, 149.07, 148.60, 142.72, 139.63, 136.05, 135.39, 135.34, 135.15, 133.30, 127.96, 125.93, 125.53, 124.13, 123.92, 119.61, 115.56, 112.31, 49.81, 21.14, 18.70 ppm. IR: ν 1652 and 3290 cm^–1^. HRMS (ESI^+^), *m*/*z* calculated for C_26_H_25_ClN_4_O_4_S [M + H]^+^ 525.1357, found 525.1359.

#### 5-Chloro-3-((3,5-dimethylphenyl)­sulfonyl)-*N*-(1-oxo-1-((1-(pyridin-4-yl)­ethyl)­amino)­propan-2-yl)-1*H*-indole-2-carboxamide (**15**)

Resynthesized as **8** starting from **21** and 1-(pyridin-4-yl)­ethanamine. Yield 50%, mp 130–133. (Lit. (23) Yield 12%, mp 130 – 133 °C). ^1^H NMR (400 MHz, DMSO-*d*_6_) δ 13.08 (s, 1H), 9.45 (d, *J* = 7.1 Hz, 1H), 8.60 (dd, *J* = 16.8 and 7.6 Hz, 1H), 8.58–8.47 (m, 2H), 8.01 (d, *J* = 2.1 Hz, 1H), 7.73–7.66 (m, 2H), 7.57 (dd, *J* = 8.8 and 4.5 Hz, 1H), 7.39–7.33 (m, 3H), 7.27 (d, *J* = 11.6 Hz, 1H), 4.95 (q, *J* = 6.8 Hz, 1H), 4.70–4.59 (m, 1H), 2.27 (d, *J* = 22.1 Hz, 6H), 1.48–1.35 (m, 6H) ppm. ^13^C NMR (100 MHz, DMSO-*d*_6_) δ 171.19, 159.08, 153.72, 150.08, 142.69, 139.62, 135.39, 127.93, 125.51, 124.13, 124.10, 121.61, 121.51, 119.55, 115.56, 60.23, 49.70, 47.95, 47.85, 22.14, 21.14, 18.75 ppm. IR: ν 1551, 1650, 2924, 3266 cm^–1^. HRMS (ESI^+^), *m*/*z* calculated for C_27_H_27_ClN_4_O_4_S [M + H]^+^ 539.1514, found 539.1515.

#### 5-Chloro-3-((3,5-dimethylphenyl)­sulfonyl)-*N*-(2-oxo-2-((1-(pyridin-4-yl)­ethyl)­amino)­ethyl)-1*H*-indole-2-carboxamide (**16**)

Resynthesized as **8** starting from **23**. Yield 55%, mp 240–242 °C. (Lit. (23) Yield 40%, mp 238–240 °C). ^1^H NMR (400 MHz, DMSO-*d*_6_) δ 13.10 (s, 1H), 9.47 (s, 1H), 8.52–8.49 (m, 3H), 7.99 (d, *J* = 2.1 Hz, 1H), 7.72 (s, 2H), 7.56 (d, *J* = 8.8 Hz, 1H), 7.36 (dd, *J* = 5.4 and 3.7 Hz, 3H), 7.27 (s, 1H), 4.99 (t, *J* = 7.2 Hz, 1H), 4.14 (d, *J* = 5.6 Hz, 2H), 2.27 (s, 6H), 1.41 (d, *J* = 7.1 Hz, 3H) ppm. ^13^C NMR (100 MHz, DMSO-*d*_6_) δ 169.76, 162.19, 151.62, 149.28, 139.61, 139.43, 134.44, 131.58, 131.51, 129.90, 128.60, 126.34, 126.14, 126.09, 122.70, 122.04, 115.58, 49.36, 43.43, 22.02, 21.14 ppm. IR: ν 1561, 1648, 2926, 3258 cm^–1^. HRMS (ESI^+^), *m*/*z* calculated for C_26_H_25_ClN_4_O_4_S [M + H]^+^ 525.1358, found 525.1361.

#### 5-Chloro-3-((3,5-dimethylphenyl)­sulfonyl)-*N*-(1-oxo-1-((pyridin-3-ylmethyl)­amino)­propan-2-yl)-1*H*-indole-2-carboxamide (**17**)

Resynthesized as **8** starting from **23**. Yield 51%, mp 275–279 °C. (Lit. (23) Yield 45%, mp 276–279 °C). ^1^H NMR (400 MHz, DMSO-*d*_6_) δ 13.19 (s, 1H), 9.47 (t, *J* = 5.7 Hz, 1H), 8.70 (t, *J* = 6.1 Hz, 1H), 8.49 (d, *J* = 5.0 Hz, 2H), 7.99 (d, *J* = 2.1 Hz, 1H), 7.72 (s, 2H), 7.57 (d, *J* = 8.8 Hz, 1H), 7.42–7.22 (m, 4H), 4.41 (d, *J* = 6.0 Hz, 2H), 4.15 (d, *J* = 5.6 Hz, 2H), 2.29 (s, 6H) ppm. ^13^C NMR (101 MHz, DMSO-*d*_6_) δ 168.94, 160.19, 149.91, 148.72, 142.92, 139.48, 135.20, 130.39, 127.90, 125.53, 124.24, 122.53, 121.98, 119.46, 115.97, 115.41, 60.17, 43.40, 41.61, and 21.14 ppm. IR: ν 1566, 1648, 2936, 3220 cm^–1^. HRMS (ESI^+^), *m*/*z* calculated for C_25_H_23_ClN_4_O_4_S [M + H]^+^ 511.1201, found 511.1201.

#### 5-Chloro-3-((3,5-dimethylphenyl)­sulfonyl)-*N*-(1-((2-nitrobenzyl)­amino)-1-oxopropan-2-yl)-1*H*-indole-2-carboxamide (**18**)

Synthesized as **8** starting from **21**. This compound was used as a crude product without any further purification.

#### 5-Chloro-3-((3,5-dimethylphenyl)­sulfonyl)-*N*-(1-((3-nitrobenzyl)­amino)-1-oxopropan-2-yl)-1*H*-indole-2-carboxamide (**19**)

Was synthesized as **8** starting from **21**. Yield 54% as white solid, mp 230–233 °C (from ethanol). ^1^H NMR (400 MHz, DMSO-*d*_6_) δ 13.05 (br s, disappeared after treatment with D_2_O, 1H), 9.45 (br d, *J* = 7.0 Hz, disappeared after treatment with D_2_O, 1H), 8.77 (br t, *J* = 6.0 Hz, disappeared after treatment with D_2_O, 1H), 8.17–8.16 (m, 1H), 8.11 (dd, *J* = 2.4 and 8.1 Hz, 2H), 8.02 (d, *J* = 2.0 Hz, 1H), 7.77 (d, *J* = 7.7 Hz, 1H), 7.68 (s, 2H), 7.62 (t, *J* = 7.9 Hz, 1H), 7.57 (d, *J* = 8.8 Hz, 1H), 7.37 (dd, *J* = 2 and 8.8 Hz, 1H), 7.26 (s, 1H), 4.65 (qn, *J* = 6.9 Hz, 1H), 4.51 (d, *J* = 6.0 Hz, 2H), 2.28 (s, 6H), 1.45 (d, J = 7.0 Hz, 3H) ppm. IR: ν 1641 and 3170 cm^–1^.

#### 5-Chloro-3-((3,5-dimethylphenyl)­sulfonyl)-*N*-(1-((4-nitrobenzyl)­amino)-1-oxopropan-2-yl)-1*H*-indole-2-carboxamide (**20**)

Synthesized as **8** starting from **21**. Yield 60% as white solid, mp >250 °C (from ethanol). ^1^H NMR (400 MHz, DMSO-*d*_6_) δ 13.07 (br s, disappeared after treatment with D_2_O, 1H), 9.46 (br d, *J* = 6.9 Hz, disappeared after treatment with D_2_O, 1H), 8.78 (br t, *J* = 6.0 Hz, disappeared after treatment with D_2_O, 1H), 8.20–8.16 (m, 2H), 8.01 (d, *J* = 2.0 Hz, 1H), 7.69 (d, *J* = 1.6 Hz, 2H), 7.57 (dd, *J* = 2.0 and 6.7 Hz, 3H), 7.36 (dd, *J* = 2.0 and 8.7 Hz, 1H), 7.26 (s, 1H), 4.64 (qn, *J* = 7.0 Hz, 1H), 4.50 (d, *J* = 6.0 Hz, 2H), 2.28 (s, 6H), 1.45 (d, *J* = 7.0 Hz, 3H) ppm. IR: ν 1640 and 3288 cm^–1^.

### General Procedure B. Preparation of Compounds **12**–**14**

#### Example. *N*-(1-((2-Aminobenzyl)­amino)-1-oxopropan-2-yl)-5-chloro-3-((3,5-dimethylphenyl)­sulfonyl)-1*H*-indole-2-carboxamide (**12**)

A solution of compound **18** (0.105 g; 0.184 mmol) and tin­(II) chloride dihydrate (0.210 g; 0.920 mmol) in ethyl acetate (2 mL) was heated under reflux for 3 h. After cooling, the reaction mixture was made basic with a saturated aqueous solution of sodium bicarbonate and filtered. The solution was then extracted with ethyl acetate. The organic layer was washed with a saturated sodium chloride solution, dried over anhydrous sodium sulfate, and filtered. After solvent evaporation, the residue was purified by column chromatography (silica gel, DCM:EtOH = 9.5:0.5), yielding product 12 (0.048 g; 48%) as white solid, mp 235–238 °C (from ethanol). ^1^H NMR (400 MHz, DMSO-*d*_6_) δ 13.07 (br s, disappeared after treatment with D_2_O, 1H), 9.41 (br d, *J* = 7.0 Hz, disappeared after treatment with D_2_O, 1H), 8.50 (br t, *J* = 6.0 Hz, disappeared after treatment with D_2_O, 1H), 8.02 (d, *J* = 2.0 Hz, 1H), 7.70 (br s, disappeared after treatment with D_2_O, 2H), 7.56 (d, *J* = 8.8 Hz, 1H), 7.35 (dd, *J* = 2.1 and 8.8 Hz, 1H), 7.27 (s, 1H), 7.01 (dd, *J* = 1.6 and 7.6 Hz, 1H), 6.96 (td, *J* = 1.6 and 7.7 Hz, 1H), 6.62 (dd, *J* = 1.2 and 8.0 Hz, 1H), 6.50 (td, *J* = 1.2 and 7.4 Hz, 1H), 5.04 (s, 2H), 4.61 (qn, *J* = 7.0 Hz, 1H), 4.20 (d, *J* = 6.0 Hz, 2H), 2.30 (s, 6H), 1.42 (d, *J* = 7.0 Hz, 3H) ppm. ^13^C NMR (100 MHz, DMSO-*d*_6_) δ 172.09, 158.81, 146.59, 142.73, 139.65, 135.99, 135.41, 133.26, 129.08, 128.30, 127.97, 125.95, 125.54, 124.13, 122.14, 119.64, 116.29, 115.54, 115.08, 112.34, 55.39, 49.78, 21.17, 18.91 ppm. IR: ν 1645 and 3296 cm^–1^. HRMS (ESI^+^), *m*/*z* calculated for C_27_H_27_ClN_4_O_4_S [M + H]^+^ 539.1514, found 539.1518.

#### *N*-(1-((3-Aminobenzyl)­amino)-1-oxopropan-2-yl)-5-chloro-3-((3,5-dimethylphenyl)­sulfonyl)-1*H*-indole-2-carboxamide (**13**)

Synthesized as **12** starting from **19**. Yield 87% as white solid, mp 166–169 °C (from ethanol). ^1^H NMR (400 MHz, DMSO-*d*_6_) δ 13.05 (br s, disappeared after treatment with D_2_O, 1H), 9.39 (br d, *J* = 8.0 Hz, disappeared after treatment with D_2_O, 1H), 8.50 (br t, *J* = 8.0 and 12.0 Hz, disappeared after treatment with D_2_O, 1H), 8.04 (d, *J* = 4.0 Hz, 1H), 7.71 (s, 2H), 7.57 (d, *J* = 8.0 Hz, 1H), 7.37 (dd, *J* = 1.1 and 7.5 Hz, 1H), 7.28 (s, 1H), 6.95 (t, *J* = 8.0 Hz, 1H), 6.49 (t, *J* = 1.0 Hz, 1H), 6.44 (dd, *J* = 2.1 and 10.0 Hz, 2H), 5.01 (br s, disappeared after treatment with D_2_O, 2H), 4.64 (qn, *J* = 9.3 Hz, 1H), 4.23–4.21 (m, 2H), 2.31 (s, 6H), 1.44 (d, *J* = 4.8 Hz, 3H) ppm. ^13^C NMR (100 MHz, DMSO-*d*_6_) δ 171.63, 158.66, 149.13, 142.75, 140.06, 139.64, 135.93, 135.41, 133.25, 129.28, 127.95, 126.01, 125.54, 124.13, 119.66, 115.55, 115.05, 113.09, 112.99, 112.36, 49.73, 42.81, 21.16, 19.09. IR: ν 1641 and 3175 cm^–1^. HRMS (ESI^+^), *m*/*z* calculated for C_27_H_27_ClN_4_O_4_S [M + H]^+^ 539.1514, found 539.1513.

#### *N*-(1-((4-Aminobenzyl)­amino)-1-oxopropan-2-yl)-5-chloro-3-((3,5-dimethylphenyl)­sulfonyl)-1*H*-indole-2-carboxamide (**14**)

Synthesized as **12** starting from **20**. Yield 47% as white solid, mp 200–203 °C (from ethanol). ^1^H NMR (400 MHz, DMSO-*d*_6_) δ 13.06 (br s, disappeared after treatment with D_2_O, 1H), 9.39 (br d, *J* = 7.2 Hz, disappeared after treatment with D_2_O, 1H), 8.42 (br t, *J* = 5.8 Hz, disappeared after treatment with D_2_O, 1H), 8.04 (d, *J* = 2.0 Hz, 1H), 7.70 (s, 2H), 7.56 (d, *J* = 8.8 Hz, 1H), 7.36 (dd, *J* = 2.0 and 8.8 Hz, 1H), 7.28 (s, 1H), 6.94 (dd, *J* = 1.9 and 6.3 Hz, 2H), 6.52–6.48 (m, 2H), 4.97 (br s, disappeared after treatment with D_2_O, 2H) 4.59 (qn, *J* = 7.1 Hz, 1H), 4.18 (dd, *J* = 1.8 and 5.6 Hz, 2H), 2.31 (s, 6H), 1.39 (d, *J* = 7.0 Hz, 3H). ^13^C NMR (100 MHz, DMSO-*d*_6_) δ 171.45, 158.67, 148.05, 142.74, 139.64, 135.97, 135.41, 133.23, 128.64, 127.96, 126.37, 125.98, 125.53, 124.14, 119.66, 115.53, 114.19, 112.35, 49.74, 42.42, 21.16, 19.00 ppm. IR: ν 1651 and 3206 cm^–1^. HRMS (ESI^+^), *m*/*z* calculated for C_27_H_27_ClN_4_O_4_S [M + H]^+^ 539.1514, found 539.1519.

### General Procedure C. Preparation of Compounds **21**–**26**, **50**, **55**, **60**, and **66**

#### Example: (5-Bromo-3-((3,5-dimethylphenyl)­sulfonyl)-1*H*-indole-2-carbonyl)­alanine (**26**)

A mixture of compound **31** (0.23 g; 0.67 mmol) was dissolved in tetrahydrofuran (THF) (62.5 mL) and water (30.5 mL). LiOH (0.048 g; 2.01 mmol) was added and the mixture was stirred at 25 °C for 2 h. THF was removed under reduced pressure, and HCl 1 N was added. The precipitate was filtered off and dried. Removal of the solvent gave compound **26** (0.197 g; 58%) as white solid, mp 205–208 °C (from ethanol). ^1^H NMR (400 MHz, DMSO-*d*_6_) δ 13.26 (br s, disappeared after treatment with D_2_O, 1H), 9.63 (br d, *J* = 7.0 Hz, disappeared after treatment with D_2_O, 1H), 8.38 (br d, *J* = 1.8 Hz, disappeared after treatment with D_2_O, 1H), 7.86 (s, 2H), 7.72–7.66 (m, 2H), 7.46 (s, 1H), 4.72 (qn, *J* = 7.1 Hz, 1H), 3.69 (s, 1H), 2.50 (s, 6H), 1.66 (d, *J* = 7.2 Hz, 3H). IR: ν 1651 and 3190 cm^–1^.

#### (5-Chloro-3-((3,5-dimethylphenyl)­sulfonyl)-1*H*-indole-2-carbonyl)­alanine (**21**)

Was synthesized as previously described.^54^

#### (5-Chloro-3-(3,5-dimethylbenzoyl)-1*H*-indole-2-carbonyl)­alanine (**22**)

Synthesized as **26** starting from **27**. Yield 64% as white solid, mp >250 °C (from ethanol). ^1^H NMR (400 MHz, DMSO-*d*_6_) δ 12.92 (br s, disappeared after treatment with D_2_O, 1H), 12.91 (br s, disappeared after treatment with D_2_O, 1H), 9.52 (br d, *J* = 6.4 Hz, disappeared after treatment with D_2_O, 1H), 7.97 (s, 1H), 7.59 (d, *J* = 9.2 Hz, 3H), 7.33 (d, *J* = 2.3 Hz, 1H), 7.30–7.29 (m, 4H), 4.12 (qn, *J* = 7.2 Hz, 1H), 2.32 (s, 6H), 1.15 (d, *J* = 6.7 Hz, 3H). IR: ν 1644 and 3189 cm^–1^.

#### (5-Chloro-3-((3,5-dimethylphenyl)­sulfonyl)-1*H*-indole-2-carbonyl)­glycine (**23**)

Was synthesized as previously described.^54^

#### (5-Chloro-3-(3,5-dichlorobenzoyl)-1*H*-indole-2-carbonyl)­alanine (**24**)

Synthesized as **26** starting from **29**. Yield 70% as white solid, mp 200–203 °C (from ethanol). ^1^H NMR (400 MHz, DMSO-*d*_6_) δ 12.96 (br s, disappeared after treatment with D_2_O, 1H), 12.71 (br s, disappeared after treatment with D_2_O, 1H), 9.27 (br d, *J* = 6.9 Hz, disappeared after treatment with D_2_O, 1H), 7.89 (t, *J* = 2.0 Hz, 1H), 7.71 (d, *J* = 2.1 Hz, 1H), 7.59 (d, *J* = 2.0 Hz, 2H), 7.57 (d, *J* = 8.7 Hz, 1H), 7.36 (dd, *J* = 2.1 and 8.7 Hz, 1H), 4.01 (qn, *J* = 7.0 Hz, 1H), 1.03 (d, *J* = 7.3 Hz, 3H). IR: ν 1648 and 3193 cm^–1^.

#### (3-Benzoyl-5-chloro-1*H*-indole-2-carbonyl)­alanine (**25**)

Synthesized as **26** starting from **30**. Yield 83% as white solid, mp 240–243 °C (from ethanol). ^1^H NMR (400 MHz, DMSO-*d*_6_) δ 12.90 (br s, disappeared after treatment with D_2_O, 1H), 12.71 (br s, disappeared after treatment with D_2_O, 1H), 9.61 (br d, *J* = 8.0 Hz, disappeared after treatment with D_2_O, 1H), 7.69–7.63 (m, 3H), 7.58 (d, *J* = 8.7 Hz, 1H), 7.52 (t, *J* = 6.3 Hz, 2H), 7.32 (dd, *J* = 2.0 and 8.6 Hz, 1H), 7.23 (d, *J* = 2.0 Hz, 1H), 4.17 (qn, *J* = 6.7 Hz, 1H), 1.16 (d, J = 8.0 Hz, 3H). IR: ν 1652 and 3201 cm^–1^.

#### (5-Chloro-3-((3,5-dichlorophenyl)­sulfonyl)-1*H*-indole-2-carbonyl)­alanine (**50**)

Synthesized as **26** starting from **49**. Yield 91% as white solid, mp 207–210 °C (from ethanol). ^1^H NMR (400 MHz, DMSO-*d*_6_) δ 13.06 (br s, disappeared after treatment with D_2_O, 1H), 13.01 (br s, disappeared after treatment with D_2_O, 1H), 9.33 (br d, *J* = 7.0 Hz, disappeared after treatment with D_2_O, 1H), 8.15 (d, *J* = 1.9 Hz, 2H), 8.02 (d, *J* = 2.0 Hz, 1H), 7.95 (t, *J* = 1.8 Hz, 1H), 7.58 (d, *J* = 8.8 Hz, 1H), 7.38 (dd, *J* = 2.0 and 8.8 Hz, 1H), 4.51 (qn, *J* = 7.4 Hz, 1H), 1.44 (d, *J* = 7.2 Hz, 3H). IR: ν 1654 and 3191 cm^–1^.

#### (3-((3,5-Dimethylphenyl)­sulfonyl)-5-methyl-1*H*-indole-2-carbonyl)­alanine (**55**)

Synthesized as **26** starting from **54**. The compound was used as a crude product without any further purification.

#### (3-((3,5-Dimethylphenyl)­sulfonyl)-5-fluoro-1*H*-indole-2-carbonyl)­alanine (**60**)

Synthesized as **26** starting from **59**. The compound was used as a crude product without any further purification.

#### (5-Chloro-3-(phenylsulfonyl)-1*H*-indole-2-carbonyl)­alanine (**66**)

Synthesized as **26** starting from **65**. The compound was used as a crude product without any further purification.

### General Procedure D. Preparation of Compounds **27**–**31**, **49**, **54**, **59**, and **65**

#### Example: Ethyl (5-Chloro-3-(3,5-dimethylbenzoyl)-1*H*-indole-2-carbonyl)­alaninate (**27**)

A mixture of **32** (0.41 g; 1.30 mmol), ethyl alaninate (0.26 g; 1.70 mmol), HATU (0.64 g; 1.70 mmol), DIPEA (0.37 g; 2.90 mmol), and DMF (18 mL) was stirred at 25 °C overnight. The solution was diluted with water and extracted with ethyl acetate. The organic layer was washed with brine, dried over anhydrous sodium sulfate, and filtered. Evaporation of the solvent gave a residue that was purified with flash chromatography to give compound **27** (0.26 g; 48%) as white solid, mp >250 °C (from ethanol). ^1^H NMR (400 MHz, DMSO-*d*_6_) δ 12.96 (br s, disappeared after treatment with D_2_O, 1H), 9.65 (br d, *J* = 5.6 Hz, disappeared after treatment with D_2_O, 1H), 7.62 (d, *J* = 8.8 Hz, 1H), 7.45 (d, *J* = 2.0 Hz, 1H), 7.40–7.34 (m, 4H), 4.17 (qn, *J* = 7.0 Hz, 3H), 2.37 (s, 6H), 1.24 (t, *J* = 6.8 Hz, 3H), 1.16 (d, *J* = 7.2 Hz, 3H). IR: ν 1641 and 3275 cm^–1^.

#### Ethyl (5-Chloro-3-((3,5-dimethylphenyl)­sulfonyl)-1*H*-indole-2-carbonyl)­alaninate (**28**)

Synthesized as previously described.^54^

#### Ethyl (5-Chloro-3-(3,5-dichlorobenzoyl)-1*H*-indole-2-carbonyl)­alaninate (**29**)

Synthesized as **27** starting from **34**. Yield 60% as white solid, mp 200–203 °C (from ethanol). ^1^H NMR (400 MHz, DMSO-*d*_6_) δ 12.98 (br s, disappeared after treatment with D_2_O, 1H), 9.32 (br d, *J* = 6.6 Hz, disappeared after treatment with D_2_O, 1H), 7.89 (t, *J* = 1.5 Hz, 1H), 7.75 (d, *J* = 1.8 Hz, 1H), 7.59 (d, *J* = 1.9 Hz, 2H), 7.57 (d, *J* = 8.8 Hz, 1H), 7.37 (dd, *J* = 2.2 and 8.6 Hz, 1H), 4.08 (q, *J* = 6.9 Hz, 2H), 4.01 (qn, *J* = 7.0 Hz, 1H), 1.18 (t, *J* = 6.9 Hz, 3H), 1.04 (d, *J* = 7.2 Hz, 3H). IR: ν 1643 and 3287 cm^–1^.

#### Ethyl (3-Benzoyl-5-chloro-1*H*-indole-2-carbonyl)­alaninate (**30**)

Synthesized as **27** starting from **35**. Yield 80% as white solid, mp 231–234 °C (from ethanol). ^1^H NMR (400 MHz, DMSO-*d*_6_) δ 12.88 (br s, disappeared after treatment with D_2_O, 1H), 9.61 (br d, *J* = 6.6 Hz, disappeared after treatment with D_2_O, 1H), 7.68–7.63 (m, 3H), 7.56 (d, *J* = 9.4 Hz, 1H), 7.52 (d, *J* = 7.6 Hz, 2H), 7.32–7.29 (m, 2H), 4.16 (qn, *J* = 7.0 Hz, 1H), 4.09 (q, *J* = 7.1 Hz, 2H), 1.18 (t, *J* = 7.1 Hz, 3H), 1.11 (d, *J* = 7.3 Hz, 3H). IR: ν 1642 and 3288 cm^–1^.

#### Ethyl (5-Bromo-3-((3,5-dimethylphenyl)­sulfonyl)-1*H*-indole-2-carbonyl)­alaninate (**31**)

Was synthesized as previously described.^55^

#### Ethyl (5-Chloro-3-((3,5-dichlorophenyl)­sulfonyl)-1*H*-indole-2-carbonyl)­alaninate (**49**)

Synthesized as **27** starting from **48**. Yield 75% as white solid, mp 196–199 °C (from ethanol). ^1^H NMR (400 MHz, DMSO-*d*_6_) δ 13.25 (br s, disappeared after treatment with D_2_O, 1H), 9.41 (br d, *J* = 6.8 Hz, disappeared after treatment with D_2_O, 1H), 8.13 (d, *J* = 1.9 Hz, 2H), 8.01 (d, *J* = 2.0 Hz, 1H), 7.95 (t, *J* = 1.9 Hz, 1H), 7.58 (d, *J* = 8.8 Hz, 1H), 7.38 (dd, *J* = 2.1 and 8.8 Hz, 1H), 4.55 (qn, *J* = 7.2 Hz, 1H), 4.19 (q, *J* = 7.1 Hz, 2H), 1.43 (d, *J* = 7.2 Hz, 3H), 1.25 (t, *J* = 7.1 Hz, 3H). IR: ν 1651 and 3290 cm^–1^.

#### Ethyl (3-((3,5-Dimethylphenyl)­sulfonyl)-5-methyl-1*H*-indole-2-carbonyl)­alaninate (**54**)

Synthesized as **27** starting from **53**. This compound was used as a crude product without any further purification.

#### Ethyl (3-((3,5-Dimethylphenyl)­sulfonyl)-5-fluoro-1*H*-indole-2-carbonyl)­alaninate (**59**)

Synthesized as **27** starting from **58**. This compound was used as a crude product without any further purification.

#### Methyl (5-Chloro-3-(phenylsulfonyl)-1*H*-indole-2-carbonyl)­alaninate (**65**)

Synthesized as **27** starting from **64**. Yield 62% as white solid, mp 223–226 °C (from ethanol). ^1^H NMR (400 MHz, DMSO-*d*_6_) δ 13.11 (br s, disappeared after treatment with D_2_O, 1H), 9.47 (br d, *J* = 6.9 Hz, disappeared after treatment with D_2_O, 1H), 8.09–8.05 (m, 2H), 8.00 (d, *J* = 2.1 Hz, 1H), 7.68–7.64 (m, 1H), 7.62–7.58 (m, 2H), 7.56 (d, *J* = 8.8 Hz, 1H), 7.37 (dd, *J* = 1.9 and 8.7 Hz, 1H), 4.61 (qn, *J* = 7.5 Hz, 1H), 3.73 (s, 3H), 1.47 (d, *J* = 7.2 Hz, 3H). IR: ν 1650 and 3292 cm^–1^.

### General Procedure E. Preparation of Compounds **32**–**36**, **48**, **53**, **58**, and **64**

#### Example: 5-Chloro-3-(3,5-dichlorobenzoyl)-1*H*-indole-2-carboxylic Acid (**34**)

A mixture of **39** (2.0 g; 6.52 mmol) in EtOH (17 mL) and NaOH (3N; 17 mL) was stirred at 80 °C for 2 h. The mixture was acidified with HCl (1 N), and the precipitate was filtered to furnish compound **34** (1.69 g; 70%) as white solid, mp 200–201 °C (from ethanol). ^1^H NMR (400 MHz, DMSO-*d*_6_) δ 12.82 (br s, disappeared after treatment with D_2_O, 1H), 7.90 (t, *J* = 2.0 Hz, 1H), 7.87 (d, *J* = 2.0 Hz, 1H), 7.69 (d, *J* = 2.0 Hz, 2H), 7.59 (d, *J* = 8.7 Hz, 1H), 7.39 (dd, *J* = 2.0 and 8.7 Hz, 1H). IR: ν 1645 and 3280 cm^–1^.

#### 5-Chloro-3-(3,5-dimethylbenzoyl)-1*H*-indole-2-carboxylic Acid (**32**)

Synthesized as previously described.^56^

#### 5-Chloro-3-((3,5-dimethylphenyl)­sulfonyl)-1*H*-indole-2-carboxylic Acid (**33**)

Synthesized as previously described.^54^

#### 5-Chloro-3-(phenylsulfonyl)-1*H*-indole-2-carboxylic Acid (**35**)

Synthesized as **34** starting from **63**. Yield 84% as white solid, mp >250 °C (from ethanol). ^1^H NMR (400 MHz, DMSO-*d*_6_) δ 13.49 (br s, disappeared after treatment with D_2_O, 1H), 12.61 (br s, disappeared after treatment with D_2_O, 1H), 7.75 (m, 2H), 7.63 (apparent tt, *J* = 1.3 and 6.8 Hz, 1H), 7.57 (d, *J* = 8.7 Hz, 1H), 7.52–7.48 (m, 3H), 7.35 (dd, *J* = 2.1 and 8.8 Hz, 1H). IR: ν 1651 and 3283 cm^–1^.

#### 5-Bromo-3-((3,5-dimethylphenyl)­sulfonyl)-1*H*-indole-2-carboxylic Acid (**36**)

Synthesized as previously described.^55^

#### 5-Chloro-3-((3,5-dichlorophenyl)­sulfonyl)-1*H*-indole-2-carboxylic Acid (**48**)

Synthesized as **34** starting from **47**. Yield 90% as white solid, mp 186–189 °C (from ethanol). ^1^H NMR (400 MHz, DMSO-*d*_6_) δ 12.20 (br s, disappeared after treatment with D_2_O, 1H), 11.55 (br s, disappeared after treatment with D_2_O, 1H), 8.08–8.06 (m, 2H), 7.85–7.83 (m, 1H), 7.60 (t, *J* = 7.9 Hz, 1H), 7.56–7.55 (m, 1H), 7.02 (s, 1H). IR: ν 1652 and 3288 cm^–1^.

#### 3-((3,5-Dimethylphenyl)­sulfonyl)-5-methyl-1*H*-indole-2-carboxylic Acid (**53**)

Synthesized as **34** starting from **52**. This compound was used as a crude product without any further purification.

#### 3-((3,5-Dimethylphenyl)­sulfonyl)-5-fluoro-1*H*-indole-2-carboxylic Acid (**58**)

Synthesized as **34** starting from **57**. This compound was used as a crude product without any further purification.

#### 5-Chloro-3-(phenylsulfonyl)-1*H*-indole-2-carboxylic Acid (**64**)

Synthesized as already described.^56^

### General Procedure F. Preparation of Compounds **38**, **42**, **47**, **52**, **57**, and **63**

#### Example. Ethyl 5-Chloro-3-((3,5-dichlorophenyl)­sulfonyl)-1*H*-indole-2-carboxylate (**47**)

*Meta*-chloroperoxybenzoic acid (mCPBA) (0.083 g; 0.50 mmol) was added to a mixture of compound 5-chloro-3-((3,5-dichlorophenyl)­thio)-1*H*-indole-2-carboxylate (**46**) (0.09 g; 0.22 mmol) in chloroform (4 mL) at 0 °C. The mixture was stirred at room temperature for 3 h, diluted with water, and extracted with chloroform. The organic layer was wahed with brine, dried over anhydrous sodium sulfate, and filtered. Removal of the solvent gave a residue that was purified by flash chromatography (Cy:ETAC = 4:1) to give product **47** (0.03 g; 32%) as white solid, mp 246–249 °C (from ethanol). ^1^H NMR (400 MHz, DMSO-*d*_6_) δ 13.47 (br s, disappeared after treatment with D_2_O, 1H), 8.24 (d, *J* = 1.9 Hz, 1H), 8.03 (d, *J* = 2.0 Hz, 2H), 7.99 (t, *J* = 1.7 Hz, 1H), 7.64 (d, *J* = 8.8 Hz, 1H), 7.47 (dd, *J* = 2.0 and 8.8 Hz, 1H), 4.39 (q, *J* = 7.5 Hz, 2H), 1.32 (t, *J* = 6.8 Hz, 3H). IR: ν 1650 and 3291 cm^–1^.

#### Ethyl 5-Chloro-3-((3,5-dimethylphenyl)­sulfonyl)-1*H*-indole-2-carboxylate (**38**)

Synthesized as previously described.^54^

#### Ethyl 5-Bromo-3-((3,5-dimethylphenyl)­sulfonyl)-1*H*-indole-2-carboxylate (**42**)

Synthesized as previously described.^55^

#### Ethyl 3-((3,5-Dimethylphenyl)­sulfonyl)-5-methyl-1*H*-indole-2-carboxylate (**52**)

Synthesized as **47** starting from **51**. This compound was used as a crude product without any further purification.

#### Ethyl 3-((3,5-Dimethylphenyl)­sulfonyl)-5-fluoro-1*H*-indole-2-carboxylate (**57**)

Synthesized as **47** starting from **56**. Yield 71% as white solid, mp 161–164 °C (from ethanol). ^1^H NMR (400 MHz, DMSO-*d*_6_) δ 12.20 (br s, disappeared after treatment with D_2_O, 1H), 11.73 (br s, disappeared after treatment with D_2_O, 1H), 7.53 (s, 1H), 7.38 (s, 1H), 7.25 (s, 1H), 7.04 (s, 1H), 6.88 (d, *J* = 2.6 Hz, 1H), 6.45 (s, 1H), 4.34 (q, *J* = 6.7 Hz, 2H), 3.81 (s, 6H), 3.75 (s, 3H). IR: ν 1657 and 3282 cm^–1^.

#### Ethyl 5-Chloro-3-(phenylsulfonyl)-1*H*-indole-2-carboxylate (**63**)

Synthesized as previously described.^56^

### General Procedure G. Preparation of Compounds **41**, **43**, **46**, **51**, **56**, and **62**

#### Example. Ethyl 5-Chloro-3-((3,5-dichlorophenyl)­thio)-1*H*-indole-2-carboxylate (**46**)

A mixture of ethyl 5-chloro-1*H*-indole-2-carboxylate (0.192 g; 0.85 mmol), compound **45** (0.390 g; 1.10 mmol), and potassium carbonate (0.175 g; 1.27 mmol) in DMSO (3 mL) was heated at 100 °C for 9 h. After cooling, the solution was diluted with water and extracted with ethyl acetate. The organic layer was washed with brine, dried over anhydrous sodium sulfate, and filtered. Removal of the solvent gave a residue that was purified by silica gel column chromatography (Cy:ETAC = 9:1) to give product **46** (0.200 g; 60%) as white solid, mp 247–250 °C (from ethanol). ^1^H NMR (400 MHz, DMSO-*d*_6_) δ 12.79 (br s, disappeared after treatment with D_2_O, 1H), 7.60 (d, *J* = 8.8 Hz, 1H), 7.52 (d, *J* = 1.9 Hz, 1H), 7.39 (dd, *J* = 2.1 and 8.8 Hz, 1H), 7.36 (d, *J* = 2.1 Hz, 1H), 7.05 (d, *J* = 1.8 Hz, 2H), 4.32 (q, *J* = 7.1 Hz, 2H), 0.93 (t, *J* = 7.1 Hz, 3H). IR: ν 1651 and 3288 cm^–1^.

#### Ethyl 5-Chloro-3-((3,5-dimethylphenyl)­thio)-1*H*-indole-2-carboxylate (**41**)

Synthesized as previously described.^54^

#### Ethyl 5-Bromo-3-((3,5-dimethylphenyl)­thio)-1*H*-indole-2-carboxylate (**43**)

Synthesized as previously described.^55^

#### Ethyl 3-((3,5-Dimethylphenyl)­thio)-5-methyl-1*H*-indole-2-carboxylate (**51**)

Synthesized as **46** starting from **44**. This compound was used as a crude product without any further purification.

#### Ethyl 3-((3,5-Dimethylphenyl)­thio)-5-fluoro-1*H*-indole-2-carboxylate (**56**)

Synthesized as **46** starting from ethyl 5-fluoro-1*H*-indole-2-carboxylate and **44**. Yield 60% as white solid, mp 188–191 °C (from ethanol). ^1^H NMR (400 MHz, DMSO-*d*_6_) δ 12.46 (br s, disappeared after treatment with D_2_O, 1H), 11.73 (br s, disappeared after treatment with D_2_O, 1H), 7.57–7.54 (m, 1H), 7.24–7.19 (m, 1H), 7.10 (d, *J* = 9.9 Hz, 1H), 6.77 (s, 1H), 6.73 (s, 2H), 4.33 (q, *J* = 6.7 Hz, 2H), 3.31 (s, 6H), 2.15 (s, 3H). IR: ν 1657 and 3282 cm^–1^.

#### Ethyl 5-Chloro-3-(phenylthio)-1*H*-indole-2-carboxylate (**62**)

Synthesized as previously described.^56^

### General Procedure H. Preparation of Compounds **44**, **45**, and **61**

#### 1,2-Bis­(3,5-dimethylphenyl)­disulfane (**44**)

Synthesized as previously described in literature https://pubs.acs.org/doi/abs/10.1021/jm00080a020.

#### 1,2-Bis­(3,5-dichlorophenyl)­disulfane (**45**)

Synthesized as previously described in literature https://pubs.acs.org/doi/abs/10.1021/jm00080a020.

#### 1,2-Diphenyldisulfane (61)

Synthesized as previously described.^56^

### General Procedure I. Preparation of Compounds **37**, **39**, and **40**

#### Example. Ethyl 5-Chloro-3-(3,5-dichlorobenzoyl)-1*H*-indole-2-carboxylate (**39**)

A mixture of aluminum chloride anhydrous (AlCl_3_) (0.715 g; 5.36 mmol), ethyl 5-chloro-1*H*-indole-2-carboxylate (1.0 g; 4.47 mmol), and 3,5-dichlorobenzoyl chloride (1.12 g; 5.36 mmol) in 1,2-dichloroethane (DCE) was placed into the MW cavity (closed dichlorobenzoyl vessel mode, PMAX 250 Pa). MW irradiation of 150 W was used, and the temperature ramped from 25 to 110 °C while stirring. Once 110 °C was reached, the reaction mixture was held for 2 min, and then cooled, diluted with water, and extracted with DCM. The organic layer was washed with brine, dried over anhydrous sodium sulfate, and filtered. Removal of the solvent gave a residue that was purified by silica gel colum chromatography (Cy:ETAC = 7:3) to give product **39** (2.0 g; 68%) as white solid, mp 205–208 °C (from ethanol). ^1^H NMR (400 MHz, DMSO-*d*_6_) δ 12.94 (br s, disappeared after treatment with D_2_O, 1H), 7.93 (t, *J* = 1.9 Hz, 1H), 7.86 (d, *J* = 1.9 Hz, 1H), 7.75 (d, *J* = 2.1 Hz, 1H), 7.71 (d, *J* = 1.9 Hz, 1H), 7.61 (d, *J* = 8.8 Hz, 1H), 7.41 (dd, *J* = 2.2 and 8.8 Hz, 1H), 4.03 (q, *J* = 7.1 Hz, 2H), 7.52–7.48 (m, 3H), 0.93 (t, *J* = 7.1 Hz, 3H). IR: ν 1654 and 3292 cm^–1^.

#### Ethyl 5-Chloro-3-(3,5-dimethylbenzoyl)-1*H*-indole-2-carboxylate (**37**)

Was synthesized as previously described.^57^

#### Ethyl 3-Benzoyl-5-chloro-1*H*-indole-2-carboxylate (**40**)

Synthesized as previously described.^58^

### Enantioselective HPLC of Compound **15**

Materials. All HPLC-grade solvents were purchased from Sigma-Aldrich (St. Louis, Missouri). Column Chiralpak IG was from Daicel (Daicel Corporation, Osaka, Japan). Experimental. Analytical HPLC analyses were carried out on a Jasco PU-980 HPLC pump equipped with a Rheodyne model 7125 (loop 20 μL) injector and coupled with a Jasco UV-975UV/VIS detector. Data were collected using the Borwin software (Jasco, Europe). Semipreparative HPLC was performed on a Waters apparatus equipped with a W1525 pump, a Rheodyne injector (loop 500 μL), and a W2487 UV detector. Analytical (250 × 4.6 mm, 3 μm) and semipreparative (250 × 10 mm, 5 μm) columns packed with the Chiralpak IG chiral stationary phase were from Daicel (Daicel Corporation, Osaka, Japan). The mobile phase was hexane/DCM/isopropanol (60/30/10) + 2% MeOH and delivered at a flow rate of 0.7 mL/min at 35 °C (or 4.0 mL/min and room temperature in semipreparative mode). The UV detector was at 300 nm. The ECD spectra of the single stereoisomer were recorded on a Jasco J710 spectropolarimeter in acetonitrile. Specific optical rotation was measured on a polarimeter Jasco P/1020 (Jasco, Europe) using a 10 cm cell.

### Inhibition of DVL1 Recruitment by FZD4

HEK293 cells were seeded in 96-well tissue culture plates at a density of 5 × 10^3^/well. After 16 h from seeding, cells were cotransfected with HA-FZD4-wt (0.04 μg/well) and GFP-tagged DVL1 (0.04 μg/well) using PEI (0.25 μL/well) in a final volume of 100 μL/well. After 24 h of transfection, the medium was replaced and supplemented with the indicated compounds diluted in culture medium. After 24 h of treatment, cells were fixed in 3.7% formaldehyde freshly diluted in PBS for 30 min at RT, quenched with 0.1 M glycine/PBS for 30 min at room temperature, and washed with PBS. Samples were observed under a Leica confocal fluorescent microscope. Inhibition of DVL recruitment was measured by counting the percentage of cells losing GFP-DVL1 localization on the plasma membrane. Experiments were performed in triplicate, and the standard deviations are indicated.

### Competition Binding Experiments

Equilibrium binding experiments were performed on a FluoroMax-4 single-photon counting spectrofluorometer (Jobin-Yvon, New Jersey, USA). The buffer used was sodium phosphate 50 mM, DMSO 20% v/v, pH 7.2, and the temperature was set at 25 °C. A constant concentration (1 μM) of a peptide mimicking the C-terminal portion of TMEM88, ranging from residue 153 to residue 159 (TSGKVWV), modified with a dansyl group covalently linked to its N-terminus, was mixed with increasing concentrations of the DVL1 PDZ domain (ranging from 2 to 38 μM). FRET emissions between 450 and 540 nm at different concentrations of the DVL1 PDZ domain were collected as averages of three independent measurements, by exciting the sample at 280 nm in a quartz cuvette with a path length of 1 cm. Experiments were performed in the absence and in the presence of 5 and 10 μM constant concentration of (*S*,*S*)-**15**. Normalized fluorescence collected at 510 nm was fitted using a hyperbolic function.

### Cell Cultures

Commercial human cell lines derived from colon carcinomas were used for the in vitro and in vivo experiments. The HCT116, DLD-1, SW480, and SW620 cells were purchased from the Interlab Cell Line Collection (IRCCS San Martino General Hospital, Genova, Italy). The SW480 and SW620 lines were obtained, respectively, from the primary tumor and a metastatic lesion of the same patient. The human chemosensitive HT29 colon cancer cells were purchased from ATCC (Manassas, Virginia). The cells were cultured in a 5% CO_2_-humidified atmosphere in the following media: RMPI-1640 (DLD-1 and HT29), McCoy’s 5A (HCT116), Leibovitz’s L-15 (SW480), and DMEM (SW620). All the media were supplemented with 10% fetal bovine serum, penicillin 100 IU/mL, streptomycin 100 μg/mL, and 2 mM l-glutamine. Human HT29/DX cells were generated by stepwise selection in media with increasing concentrations of DOX, as described previously,^53^ and maintained in culture medium with a final concentration of 200 nM DOX. All cell lines were authenticated by microsatellite analysis, using the PowerPlex kit (Promega Corporation, Madison, Wisconsin; last authentication: January 2022).

### Proliferation Assay

Adherent cell cultures were split, counted, and seeded in 96-well plates (2000–5000 cells/well). The next day, cells were treated with increasing doses, from 1 to 500 μM, of (*S*)-**1** or (*S*,*S*)-**15**, or the vehicle alone (DMSO), for 72 h. Similar effects were obtained regardless of whether the culture media were replaced every 24 h or left unchanged throughout the incubation period, suggesting compound stability under assay conditions. Finally, 10 mL of tetrazolium salt WST-1 (5-(2,4-disulfophenyl)-2-(4-iodophenyl)-3-(4-nitrophenyl)-2*H*-tetrazolium, inner salt, monosodium salt) was added to 100 μL culture medium in each well, and the absorbance was read 3 h later at 450 nm using a microplate ELISA reader (Tecan Group Ltd., Männedorf, Switzerland). The experiments were performed in quadruplicate, and IC_50_ values were calculated for each cell line by using the AAT Bioquest online tool.^36^

### Apoptosis Assay

The Cell Death Detection ELISAPLUS kit (Roche Diagnostics, Monza, Italy) was used to quantify apoptosis induction in SW620, SW480, HCT116, and DLD-1 cell lines. Cells were seeded in triplicate in 96-well culture plates (10,000 cells/well) and treated the next day with 50 μM (*S*)-**1** or (*S*,*S*)-**15**, or with an equal volume of DMSO. After 24 h of incubation, cells were centrifuged to remove the supernatant, the pellets were resuspended in 200 μL of lysis buffer and then analyzed for the presence of cytoplasmic histone-associated DNA fragments according to the manufacturer’s instructions. Briefly, lysates were added in triplicate to the wells of a streptavidin-coated microplate, together with a mixture of biotinylated antihistone and anti-DNA peroxidase-conjugated antibodies. The plate was incubated for 2 h and then washed, and the ABTS (2,2′-azino-bis­[3-ethylbenzothiazoline-6-sulfonic acid]) substrate was added. Absorbance was determined photometrically at 450 nm (with reference wavelength of 495 nm), and the specific enrichment of mono- and oligonucleosomes released into the cytoplasm was calculated as the ratio: mU (treated cells)/mU (control cells), where mU = absorbance × [10^–3^].

### Expression of β-Catenin in HCT116 Cells

Cells were seeded in a six-well plate and incubated until 80% confluent. Then, cells were were treated with (*S*,*S*)-**15**, **67**, the combination (*S*,*S*)-**15** and **67**, or an equal volume of DMSO as control for 24 h. Compounds (*S*,*S*)-**15** and **67** were used at 23 μM, a dose that closely approximates their IC_50_ concentration, in both single and combination studies. At the end of the incubation, cells were collected and used for total RNA or protein extraction as described below.

### RNA Extraction and Quantitative Real-Time Reverse Transcription PCR (qRT-PCR)

The TRIzol (Invitrogen Life Technologies, Carlsbad, California, USA) was used to perform RNA extraction from both cultured cells and xenograft tissues following the manufacturer’s instructions. Tissue samples were disrupted using an Ultra-Turrax homogenizer (IKA, Staufen, Germany), adapting the TRIzol volume to the sample weight. DNase I (Sigma-Aldrich) was used to eliminate DNA contamination, and the total RNA concentration was determined by a NanoDrop 1000 spectrophotometer (Nanodrop Technologies, Wilmington, Delaware, USA). The MMLV reverse transcriptase (Promega, Madison, Wisconsin, USA) was used to reverse transcribe 1–2 μg of total RNA with oligo­(dT)­18 as primer. The resulting cDNAs were subjected to qPCR using a RealPlex4 real-time PCR detection system (Eppendorf Co., Ltd, Hamburg, Germany), the SYBR Green Real-Time PCR Master Mix (Toyobo (Shanghai) Biotech Co., Ltd., Shanghai, China), and specific primers. The qPCR protocol included 40 cycles of denaturation for 15 s at 95 °C, annealing for 30 s at 58 °C, and extension for 42 s at 72 °C. The 2^–ΔΔCt^ method was used to measure expression levels of the target gene relative to the 18S rRNA reference gene.

### Protein Extraction and Western Blotting

Cells were lysed in RIPA buffer supplemented with fresh protease and phosphatase inhibitor cocktails. Tissue samples were disrupted using an Ultra-Turrax homogenizer (IKA, Staufen, Germany), adjusting the buffer volume to the sample weight. Lysates were sonicated and centrifuged at 12,000*g* for 15 min at 4 °C; then, supernatants were recovered, and protein concentrations were determined by the Bradford assay. 30 μg of protein was mixed with Laemmli buffer containing 0.2% β-mercaptoethanol, heated at 95 °C for 5 min, and then separated by sodium dodecyl sulfate-polyacrylamide gel electrophoresis (SDS-PAGE). Proteins were transferred onto polyvinylidene fluoride (PVDF) membranes (Millipore, Billerica, Massachusetts, USA) with the Bio-Rad Mini Trans-Blot Cell system. After blocking and washing, membranes were incubated with primary antibodies anti-β-catenin D10A8 XP Rabbit mAb #8480, anti-p-β-catenin (Ser675) D2F1 XP Rabbit mAb #68076, and anti-GAPDH 14C10 Rabbit mAb #2118, Cell Signaling Technology, Massachusetts, USA) at 37 °C for 45 min. The membranes were carefully washed and then incubated with appropriate secondary antibodies (Antirabbit IgG, HRP-linked Antibody (#7074), Cell Signaling Technology, Massachusetts, USA) at 37 °C for 45 min. The membranes were washed four times with Tris-buffered saline-Tween 20 (TBST) at room temperature and finally treated with the ECL-enhanced chemiluminescence substrate (ECL kit, Pierce Biotechnology, Rockford, Illinois, USA).

### Immunofluorescence Staining

Fresh tissues were immersed in 4% paraformaldehyde (Sigma-Aldrich) for fixation at room temperature for 30 min. The tissues were then dehydrated in an ethanol gradient, embedded in paraffin, sectioned (thickness: 6 μm), and immersed in xylene for dewaxing. Tissue sections were blocked with immunohistochemical blocking solution (Beyotime Biotechnology Co., Ltd., Zhejiang, China) at 37 °C for 30 min. The blocking solution was then discarded, and the sections were washed three times for 5 min each with immunohistochemical washing solution (Beyotime Biotechnology). Then, the primary antibody anti-β-catenin (D10A8) XP rabbit mAb (#8480, Cell Signaling Technology, Massachusetts, USA) or antirabbit Ki67 antibody (ab15580, Abcam, Massachusetts, USA) was added and sections were incubated at 37 °C for 45 min. The sections were washed three times for 5 min each with immunohistochemical washing solution (Beyotime Biotechnology). Subsequently, the Alexa Fluor 555-conjugated goat antirabbit IgG H&L secondary antibody (Abcam, Massachusetts, USA) was added, and the tissues were incubated at 37 °C for 45 min. The antibody solution was discarded, and the sections were washed three times for 5 min each with immunohistochemical washing solution (Beyotime Biotechnology). Cell nuclei were counterstained with DAPI (Sigma-Aldrich) for 5 min at room temperature, and slides were coverslipped using an immunofluorescence mounting medium (Sigma-Aldrich).

### In Vivo Xenograft Experiments

Briefly, 10-week-old male BALB/C^nu/nu^ mice (*n* = 16) were purchased from the Shanghai University of Traditional Chinese Medicine following Institutional Animal Care and Use Committee approval in accordance with institutional guidelines. All animal experiments were performed following the protocols evaluated and approved by the Ethics Committee for Animal Experiments of Shanghai Geriatric Institute of Chinese Medicine (Ethics Approval Number: SHAGE-AE-2025004). HCT116 cells at the logarithmic growth phase were harvested, and 1 × 10^8^ cells/mL were inoculated subcutaneously into the mice. After tumor establishment, the mice were randomly divided into four groups of four animals each. Group #1 received 100 μL of (*S*,*S*)-**15** (25 mg/kg), group #2 23 mg/kg of 5-FU, group #3 a drug combination of (*S*,*S*)-**15** at 14 mg/kg and 5-FU at 12 mg/kg, and group #4 100 μL of saline, by intraperitoneal injection (IP) every 2 days. After 40 days, the mice were sacrificed and the tumors were excised and weighed and their volumes calculated using the following formula:

$$\mathrm{Tumor}\;\mathrm{volume}\left({\mathrm{cm}}^{3}\right)=\left({\mathrm{ab}}^{2}\right)/2\left(a:\mathrm{the}\;\mathrm{longest}\;\mathrm{axis}\left(\mathrm{cm}\right),b:\mathrm{the}\;\mathrm{shortest}\;\mathrm{axis}\left(\mathrm{cm}\right)\right)$$

### Hematoxylin and Eosin Staining

Tissue samples were fixed in 4% paraformaldehyde, dehydrated, and embedded in paraffin. The paraffin-embedded tissue blocks were cut into 4 μm sections using a microtome, and the sections were mounted onto glass slides. Subsequently, the sections were dewaxed using xylene and rehydrated through a graded ethanol series. The sections were stained with hematoxylin (H) for 5 min at room temperature, and then 1% ethanol was added for 30 s for differentiation. Afterward, aqueous ammonia was added for 1 min for blueing, followed by rinsing in distilled water for 5 min. The sections were stained with eosin (E) for 2 min at room temperature and then rinsed with distilled water for 2 min. Then, the sections were dehydrated through a graded ethanol series, and xylene was added for 2 min for clearing. Finally, the sections were sealed and mounted with neutral resin.

### In Vitro Oxidative Metabolic Stability: Intrinsic Clearance in Microsomes^59^

Mouse (Sigma-Aldrich, CD1 male, pooled) and human (Sigma-Aldrich, human, pooled) microsomes at 0.5 mg/mL were preincubated for 10 min at 37 °C with the test compound **12** dissolved in DMSO at 1 μM in 50 mM phosphate buffer (pH 7.4) containing 3 mM MgCl_2_. The reaction was then started by adding the cofactor mixture solution (NADP, glucose-6-phosphate, and glucose-6-phosphate dehydrogenase in 2% NaHCO_3_). The samples were taken at 0, 10, 30, 45, and 60 min and added to acetonitrile to stop the reaction. Samples were then centrifuged, and the supernatants were analyzed by LC-MS/MS to quantify the amount of compound. A control sample without cofactor was always added to check the chemical stability of the test compound. Two reference compounds of known metabolic stability, propranolol and verapamil, were always used as controls. A fixed concentration of verapamil was added in every sample as an internal standard for LC-MS/MS analyses. The percentage of the area of the test compound remaining at the various incubation times was calculated with respect to the area of the compound at time 0 min.

The intrinsic clearance (Cli) was calculated by the equation:

Cli(μL/min/mg)=k/microsomalconc.x1000

where *k* is the rate constant (min^–1^); microsomal protein conc. = 0.5 mg protein/mL. The rate constant, *k* (min^–1^) derived from the exponential decay equation (peak area/IS vs time), was used to calculate the rate of Cli. Classification of in vitro stability is presented in Table 6.

### LC–MS/MS Analytical Method

Samples were analyzed under the following conditions: UPLC Waters coupled with an API 3200 triple-quadrupole (ABSciex); eluents, (phase A) 95% water, 5% acetonitrile + 0.1% HCOOH, (phase B) 5% water, 95% acetonitrile + 0.1% HCOOH; flow rate, 0.3 mL/min; column, Gemini-Nx 5 μm C18 110A (50 × 2.00 mm) at 35 °C; injection volume, 10 μL. Source conditions ESI positive: T 400 °C, Gas 1 30, Gas 2 35, CUR 30, IS 5500, CAD 5.

### Doxorubicin Accumulation and P-gp Activity

The intracellular accumulation of doxorubicin was measured fluorimetrically.^53^ Cells were incubated for 3 h with 5 μM doxorubicin, alone or with (*S*,*S*)-**15** at the following concentrations: 1, 10, or 100 nM. After two washing steps in PBS, cells were trypsinized, centrifuged at 13,000 × *g* for 5 min, and resuspended in 0.5 mL of 1/1 solution ethanol/0.3 N HCl. A 50 μL aliquot was sonicated and used to quantify the intracellular protein content. In the remaining sample, the intracellular fluorescence of doxorubicin was measured with a Synergy HTX microplate reader (excitation wavelength: 475 nm; emission wavelength: 553 nm). Fluorescence was converted into nmol/mg cell proteins, using a calibration curve previously set. The activity of P-gp was measured spectrophotometrically as rate of ATP hydrolysis,^53^ as previously reported.^60^

### Pharmacokinetic Parameters

Male CD1 mice (3 + 3) of 25–30 g weight were provided by Envigo, Italy. Animals were acclimatized for about 5 days before the treatment. IV formulation: (*S,S*)-**15** at a concentration of 0.2 mg/mL in PBS containing 5% DMSO. To prepare 10 mL of IV formulation, 2 mg of (*S*,*S*)-**15** in a vial was reconstituted with 0.5 mL of DMSO and vortexed. After complete dissolution of the test compound, 9.5 mL of PBS was added. OS formulation: (*S*,*S*)-**15** at a concentration of 0.5 mg/mL in PBS containing 5% DMSO. To prepare 10 mL of OS formulation, 5 mg of (*S*,*S*)-**15** in a vial was reconstituted with 0.5 mL of DMSO and vortexed. After complete dissolution of the test compound, 9.5 mL of PBS was added. Sampling time points were scheduled for IV at 5 min, 20 min, 1 h, 2 h, 4 h, and 7 h, and for OS at 15 min, 30 min, 1 h, 2 h, 4 h, and 7 h. Target plasma volumes of 50 μL were collected from abdominal aorta under deep gaseous anesthesia. At study termination, the animals were sacrificed by exsanguination under anesthesia.

### PK Sample Analysis

25 μL of plasma was added to 150 μL of acetonitrile containing verapamil as internal standard (0.1 μM) (WS_IS) in a 96-well plate. Samples were shaken for 15 min at 350 rpm at room temperature and then centrifuged for 15 min at 5 °C at 4600 rpm. Samples were then transferred to a 96-well plate for analysis. Working solutions (WS) for the calibration curves and quality control (QC) samples were prepared by serial dilution in acetonitrile over the concentration range 50,000–5 ng/mL. Calibration curves were prepared in each matrix by adding 2.5 μL of WSs to 22.5 μL of blank matrix. Samples were prepared on ice, immediately added to 150 μL of acetonitrile with IS (WS_IS), and processed as described above.

### LC Methods

Samples were analyzed on an Acquity UPLC (Waters) coupled with an AB Sciex API 3200 Triple Quadrupole: mobile phase A, water containing 0.1% formic acid; mobile phase B: acetonitrile containing 0.1% formic acid; injection volume 5 μL - Kinetex 2.6 μm Biphenyl 100A 100 × 3 mm; gradient, flow 0.4 mL/min (Table 2S, Supporting Information). MS parameters are reported in Table 3S, Supporting Information.

### Molecular Modeling

Docking calculations were performed on an Intel Xeon Silver-powered machine running Ubuntu 20.04 LTS. The PDZ domain of DVL1 was generated by homology modeling using experimentally resolved PDZ structures of DVL2 as templates (PDB IDs: 3CBZ, 3CBY, and 3CC0).^61^ The amino acid sequence of DVL1 was retrieved from the UniProt database (UniProt ID: O14640; https://www.uniprot.org/uniprotkb/O14640/entry). Sequence alignment revealed 75% identity and 85% similarity between the target and template sequences. Protein structures were prepared using the Maestro Protein Preparation Wizard,^62,63^ where hydrogen atoms were added and the system was energy-minimized with all heavy atoms restrained until a convergence threshold of 0.05 kcal·mol^–1^·Å^–1^ was reached. Ligand structures were protonated at physiological pH and minimized using the OPLS4 force field under the same convergence criteria. Docking simulations were carried out with PLANTS,^64^ defining a binding site sphere with a radius of 12 Å and using default docking parameters. Pictures were generated with PyMOL.^65^

### Statistical Analysis

Each experiment was performed at least three times, and the values are reported as the mean ± standard deviation, where applicable. The Student’s *t* test was used to evaluate differences between two independent groups, while one-way ANOVA followed by Tukey’s posthoc test was applied for comparisons among three or more groups. A *p*-value <0.05 was considered statistically significant.

## Supplementary Material









## Notes

Molecular formula strings with the associated biological data (CSV)
DVL homology model (PDB)
DVL/**15** model (PDB)
(Figure 1S) Enantioselective HPLC control of (*S*,*S*)-**15** stereoisomer after enantioselective synthesis; (Figure 2S) inhibition of SW620, SW480, HCT116, and DLD-1 cell growth by compounds (*S*)-**1**; (Figure 3S) structure of compound **67**; (Figure 4S) selected examples of tumor tissues collected from the backs of the mice; (Table 1S) compound MRM transitions and conditions; (Table 2S) chromatographic gradient; and (Table 3S) MS parameters ESI positive (PDF)

## Abbreviations

aa: amino acid
ADME: Absorption, Distribution, Metabolism, Excretion
CRC: colorectal cancer
Ctrl: control
Cy: cyclohexane
DCM: dichloromethane
DIPEA: *N*,*N*-diisopropylethylamine
DMSO: dimethyl sulfoxide
DOX: doxorubicin
DVL: Dishevelled (Dsh)
ETAC: ethyl acetate
5-FU: 5-fluorouracil
FZ: Frizzled
GIA: gastrointestinal absorption
HATU: 1-[bis­(dimethylamino)­methylene]-1*H*-1,2,3-triazolo­[4,5-*b*]­pyridinium 3-oxid hexafluorophosphate
IV: intravenous administration
LEF: lymphoid enhancer-binding factor
MDR: multidrug-resistant
MRT: mean residence time
P-gp: P-glycoprotein
p-β-catenin: phospho-β-catenin
PBS: phosphate-buffered saline
PDZ: postsynaptic density 95/disc large/zonula occludens-1
saline: isotonic 0.9% sodium chloride solution
SAR: structure–activity relationship
TCF: T-cell factor
THF: tetrahydrofuran
Wnt: Wingless/integrase-1
XOS: oral administration.
