# Supplementary material for: (S,S)‑5-Chloro-3-((3,5-dimethylphenyl)sulfonyl)‑N‑(1-oxo-1-((1-(pyridin-4-yl)ethyl)amino)propan-2-yl)‑1H‑indole-2-carboxamide, a New Dishevelled 1 and P‑Glycoprotein Dual Inhibitor as a Anticancer Agent
Source: J Med Chem. 2026 Jun 12;69(12):14667–90. doi: 10.1021/acs.jmedchem.6c00706 (PMC13312410; doi:10.1021/acs.jmedchem.6c00706)
Supplement: Supplementary file 4 [file jm6c00706_si_004.pdf]

Supporting Information

(*S,S*)-5-Chloro-3-((3,5-dimethylphenyl)sulfonyl)-N-(1-oxo-1-((1-(pyridin-4-yl)ethyl)amino)propan-2-yl)-1H-indole-2-carboxamide, a New Dishevelled 1 and P-glycoprotein Dual Inhibitor as Anticancer Agent

Michela Puxeddu,[a,+] Zeyu Cui,[b,+] Claudia Colla,[a] Marianna Nalli,[a] Simone Manetto,[a] Alessia Ciogli,[a] Petra Cuřínová,[c] Marianna Bufano,[c] Angelo Toto,[d] Stefano Gianni,[d] Arianna Pastore,[e] Mariano Stornaiuolo,[e] Enke Baldini,[f] Salvatore Ulisse,[f] Joanna Kopecka,[g] Chiara Riganti,[g] Chiara Bigogno,[h] Giulio Dondio,[h] Te Liu,[b,*] Antonio Coluccia,[a] Giuseppe La Regina,[a] and Romano Silvestri,[a,*]

[a] Laboratory Affiliated with the Institute Pasteur Italy - Cenci Bolognetti Foundation, Department of Drug Chemistry and Technologies, Sapienza University of Rome, Piazzale Aldo Moro 5, 00185 Rome, Italy.
[b] Shanghai Geriatric Institute of Chinese Medicine, Shanghai University of Traditional Chinese Medicine, 365 South Xiangyang Road, Shanghai 200031, China.
[c] Department of Organic Chemistry, University of Chemistry and Technology Prague, Technická 5, Prague 6, 16628, Czech Republic.
[d] Laboratory Affiliated with the Institute Pasteur Italy - Cenci Bolognetti Foundation, Biochemical Sciences "Rossi Fanelli", Institute of Biology and Molecular Pathology of CNR, Sapienza Università di Roma, Piazzale Aldo Moro 5, 00185 Rome, Italy
[e] Department of Pharmacy, University of Naples "Federico II", Via Domenico Montesano, 49, 80131 Naples, Italy
[f] Department of Surgery, Sapienza University of Rome, Viale Regina Elena 324, 00185 Rome, Italy
[g] Department of Oncology and Molecular Biotecnology Center "Guido Tarone", Via Nizza 44, 10126 Turin, Italy
[h] Aphad SrL, Via della Resistenza 65, 20090 Buccinasco, Italy.

Corresponding authors:
*Te Liu, e-mail: liute1979@shutcm.edu.cn; *Romano Silvestri, e-mail: romano.silvestri@uniroma1.it

Contents of the SI

- Figure 1S. Enantioselective HPLC control of (*S,S*)-15 stereoisomer after enantioselective synthesis
- Figure 2S. Inhibition of SW620, SW480, HCT116 and DLD-1 cells by compound (*S*)-1
- Figure 3S. Structure of compound 67
- Figure 4S. Examples #2-#4 of tumor tissues collected from the backs of the mice
- Table 1S. Compound MRM transitions and conditions.

- Table 2S. Chromatographic gradient
- Table 3S. MS parameters ESI positive

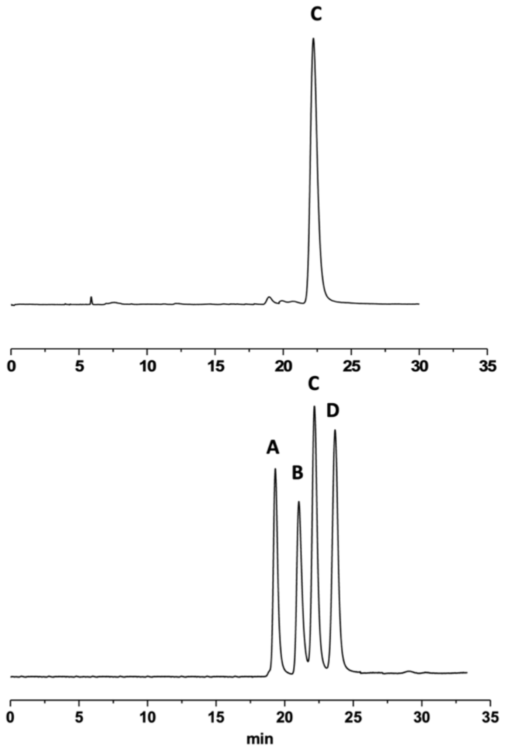

Figure 1S. Enantioselective HPLC control of (*S,S*)-**15** stereoisomer after enantioselective synthesis. Calculated enantiomeric excess (% e.e) of the four fractions: A, 99.02%; B, ≥99.99%; C, 98.04; D, 98.10.

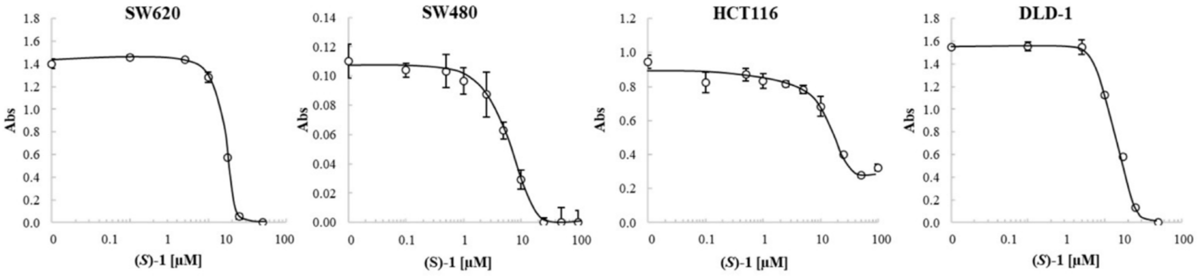

Figure 2S. Growth inhibition of SW620, SW480, HCT116 and DLD-1 colon cancer cells treated with increasing doses of compound (*S*)-**1** (1-500 μM) or the vehicle alone (DMSO). Data are presented as mean ± SD (n = 4).

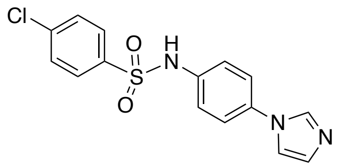

Figure 3S. Structure cof compound **67.**

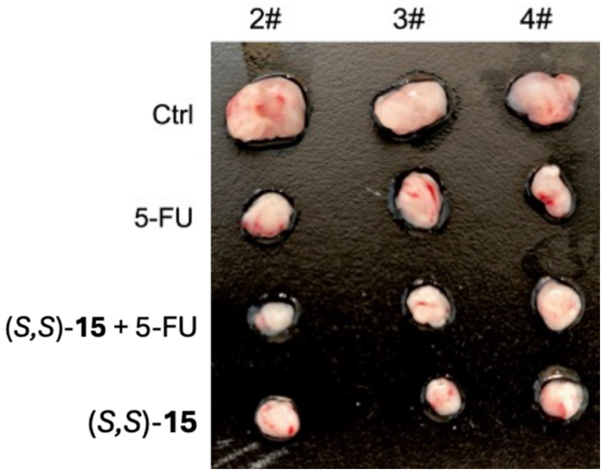

Figure 4S. Examples #2-#4 of tumor tissues collected from the backs of the mice treated with saline, (*S,S*)-**15** (25 mg/kg), 5-FU (23 mg/kg) or 1:1 (*S,S*)-**15** (14 mg/kg) + 5-FU (12 mg/kg) drug combination.

Table 1S. Compound MRM transitions and conditions.

Compound	Parent Ion[a]	Product Ion[b]	DP (V)[c]	CE (eV)[d]
7-EC	190.9	163.0	56	23
Verapamil	455.4	165.1	31	35
(*S*)-**1**	525.3	346.1	62	41
(*S,S*)-**15**	539.3	166.2	62	63

[a]Ion of the entire molecule. [b]Fragment generated from the parent ion. Declustering Potential (V) applied to the ion source. [d] Collision Energy, energy (eV) passed to the parent ion in the collision cell.

Table 2S. Chromatographic gradient

Time (min)	%B
0	5
1	5
2	95
4	95
4.5	5
5.5	5

Table 3S. MS parameters ESI positive

Compd	Parent Ion (Q1)	Product Ion (Q3)	Dwell Time ms	Declustering Potential (EV)	Entrance Potential (V)	Collision Energy (ev)	Cell Exit Potential (V)
(S,S)-**15**_1	539.3	166.2	150	62	9	63	3
(S,S)-**15**_2	539.3	346.4	150	65	7	43	5
(S,S)-**15**_3	539.3	123.3	150	63	7	32	1
(S,S)-**15**_4	539.3	389	150	66	9	34	7
Verapamil IS[a]	455.374	165.31	150	31	10	35	4

Parameters	CUR	CAD	TEMP C	IS	GS1	GS2
	30	5	450	5500	45	45

[a]IS: internal standard
